# Comparative efficacy of different doses of mesenchymal stem cells derived from different tissue sources for knee osteoarthritis: a systematic review and network meta-analysis of randomized controlled trials

**DOI:** 10.7717/peerj.20776

**Published:** 2026-03-09

**Authors:** Ruimou Xie, Jingyang Yu, Yutong Feng, Yanlin Zhang, Xueyi Ni, Hainan Jin, Yu Pan

**Affiliations:** 1 Department of Rehabilitation Medicine, Beijing Tsinghua Changgung Hospital, School of Clinical Medicine, Tsinghua Medicine, Tsinghua University, Beijing, China; 2 Division of Surgery and Interventional Science, University College London, London, United Kingdom

**Keywords:** Knee osteoarthritis, Mesenchymal stem cells, Cell dosage, Cell source, Network meta-analysis

## Abstract

**Introduction:**

Knee osteoarthritis (KOA) remains a leading cause of disability, and mesenchymal stem cells (MSCs) show potential for KOA treatment. However, existing studies demonstrate conflicting results on the optimal dose and tissue source of MSCs for KOA treatment. This gap limits evidence-based treatment decisions.

**Materials & Methods:**

A network meta-analysis (NMA) was conducted to evaluate the efficacy and safety of MSCs at different doses and from tissue sources in treating KOA. Randomized controlled trials (RCTs) were searched up to November 12, 2025. MSC doses were categorized into low ((0 < 20) ×10^6^), moderate ((20 ≤ cells < 50) ×10^6^), and high (cells ≥ 50 ×10^6^). Efficacy was assessed using Visual Analog Scale (VAS) scores, Western Ontario and McMaster Universities Arthritis Index (WOMAC) scores, and adverse events (AEs) at 3, 6, and 12 months.

**Results:**

A total of 11 RCTs were included. Except for moderate-dose bone-derived MSCs (M_BMSCS), all treatment groups significantly improved VAS scores at 3, 6, and 12 months. High-dose adipose-derived MSCs (H_ADSCS) showed superior efficacy at 3 months, while moderate-dose adipose-derived MSCs (M_ADSCS) was most effective at 6 and 12 months. For WOMAC scores, significant improvements were seen at 12 months for high-dose, moderate-dose, and low-dose adipose-derived MSCs (ADSCS), as well as low-dose bone-derived MSCs (L_BMSCS), with M_ADSCS showing the greatest benefit. Low-dose ADSCS (L_ADSCS) had a significantly low risk of AEs.

**Discussion:**

Eleven RCTs involving 602 participants were analyzed. M_ADSCS showed the greatest long-term improvements in pain (VAS) and joint function (WOMAC), with balanced efficacy and safety. High-dose ADSCS provided superior short-term analgesia but induced more adverse events, likely due to cell overcrowding and microenvironmental stress. Molecular evidence suggests that ADSCS exert stronger anti-inflammatory and immunomodulatory effects than MSCs from other sources. Variability in dose-response relationships indicates that better MSC dosing may depend on disease severity and patient factors, such as BMI and comorbidities. Further large-scale, standardized RCTs are needed to refine dosing strategies and optimize MSC therapy for KOA.

**Conclusions:**

This NMA identifies M_ADSCS as the most effective MSC treatment for KOA, with balanced efficacy and safety profiles. These findings provide valuable insights for optimizing MSC therapy and highlight the need for further research on dosing and tissue sources.

## Introduction

Knee osteoarthritis (KOA) is a prevalent degenerative condition characterized by deterioration of articular cartilage, subchondral sclerosis, and bony outgrowths at the edges of the joint (Zhang et al., 2023). The prevalence of KOA in adults aged ≥50 years ranges from 14% to 38% in females and 4% to 14% in males (Katz, Arant & Loeser, 2021). Currently, in addition to regular methods, such as strength exercises, aerobic exercises, and weight loss, clinical treatments of KOA are restricted to symptomatic management, such as non-steroidal anti-inflammatory medicines (showing limited efficacy) and knee arthroplasty when KOA reaches the mid-late stage.

Recently, intra-articular injection of mesenchymal stem/stromal cells (MSCs), also called mesenchymal progenitor cells, has gathered much attention due to their ability to modulate the immune system, providing promising therapeutic potential for KOA (Furia et al., 2022). MSCs can exert immunoregulatory effects by releasing a diverse range of cytokines and other growth factors (Higgins et al., 2003). Additionally, MSCs and their secretomes can secrete and upregulate tissue inhibitors of metalloproteinases (TIMPs), which counteract excessive matrix metalloproteinase activity in KOA progression (Lozito & Tuan, 2011). MSCs with sustained high TIMP-1 secretion exhibit strong trophic repair capacity in osteoarthritic environments, underscoring TIMP-mediated matrix regulation as an important mechanism of MSC therapy (Salerno et al., 2020).

MSCs that are commonly used in clinical trials of KOA include adipose-derived MSCs (ADSCS), bone marrow MSCs (BMSCS), and umbilical cord MSCs (UCSCS).

Several clinical trials have evaluated the efficacy of MSCs from different sources in the management of KOA. Sadri et al. (2023) demonstrated that ADSCS (100 ×10^6^) significantly decreased Visual Analog Scale (VAS) scores during the 12-month follow-up compared to the control treatment of normal saline. Emadedin et al. (2018) reported that the intra-articular injection of 40 × 10^6^ BMSCS could significantly improve Western Ontario and McMaster Universities Arthritis Index (WOMAC) total scores in KOA patients at 3 and 6 months following the injection compared with the control group. However, the source of MSCs used in current clinical trials is distinct, and the dose used varies from 2.5 × 10^6^ to 100 × 10^6^ (Kabat et al., 2019; Shariatzadeh, Song & Wilson, 2019).

Therefore, this study aimed to identify the optimal source and dose of MSCs for KOA treatment. Due to the lack of direct comparative evidence on different sources and doses of MSCs, the available evidence was indirectly compared using Bayesian network meta-analysis (NMA) to provide evidence for clinical practice.

## Methods

### Research proposal and enrollment

This systematic review and NMA followed the PRISMA guidelines 2020 (Page et al., 2021). The study protocol has been registered in the PROSPERO (CRD42024543254).

### Inclusion criteria and exclusion criteria

The inclusion and exclusion criteria adhered to the Participants, Intervention, Comparison, Outcome, and Study (PICOS) principle. The inclusion criteria were as follows: (1) All trials fulfilled the diagnostic criteria for primary adult KOA, regardless of the individual’s sex or race. (2) The patients in the experiment group only received intra-articular injections of MSCs into the knee joint. Those who had received combined injections of MSCs and other effective drugs were excluded. (3) Adults with radiographically confirmed KOA of Kellgren–Lawrence (K–L) grade II–IV were included. (4) The MSCs used were either allogeneic or autologous and were isolated from human adipose tissue, bone marrow, placenta, or synovium. (5) The patients in the control group only received standard intra-articular treatment with either hyaluronic acid or normal saline; no other active comparators, such as platelet-rich plasma, arthroscopic procedures, or high tibial osteotomy, were included. (6) The article mentioned at least one of the following outcomes: VAS, WOMAC, and adverse events (AEs). (7) Only randomized controlled trials (RCTs) in English or Chinese language were included, without any limitations on the publication year.

The exclusion criteria were as follows: (1) RCTs did not mention the dose of MSCs used for KOA treatment. (2) Substandard articles, redundant publication, and insufficient data. (3) Evaluations, case studies, conference papers, and research conducted on animals.

### Search strategy

PubMed, EMBASE, Web of Science, Cochrane Library, and China National Knowledge Infrastructure were searched independently by two authors (Jingyang Yu and Ruimou Xie) from inception to November 12, 2025. An inclusive search strategy was designed to capture all MSC sources with potential clinical use in KOA (*e.g.*, adipose, bone marrow, placental, and synovium-derived MSCs). Both English and Chinese publications were included. The search combined subject headings with free-text terms and is detailed in Supplementary Appendix 1. Disagreements between the reviewers were resolved through discussion; if consensus could not be reached, the issue was referred to a third senior reviewer (Yu Pan), who independently assessed the study and made the final decision.

### Literature screening and data extraction

Two researchers independently evaluated the studies according to the predefined criteria. Duplicates were first removed in EndNote. The titles and abstracts of the remaining articles were scanned, and mismatches were eliminated. Afterward, the full texts of the remaining papers were thoroughly examined. Disputes were settled by discussion with a third reviewer. Two reviewers independently extracted the data from each enrolled RCT using a standardized method, including study parameters (author, nation, and publication date), patient characteristics (sample size, Body Mass Index (BMI), sex, K-L grade, and mean age), information on cell dosages, intervention strategies, and outcome indicators. Literature screening was performed from November 15 to November 25, 2025, and data extraction started from November 26 to December 5, 2025.

Notably, due to the time-sensitive nature of the research and the need to ensure timely review, search results were screened based on eligibility criteria before PROSPERO registration. At that time, the protocol had already been developed in detail, and the initial screening was initiated to keep the research progressing during PROSPERO submission. Importantly, the review protocol remained unchanged during literature screening and data extraction, and full transparency has been maintained in documenting the methodology. The PROSPERO registration was submitted as soon as possible to ensure a public record of the planned methodology.

### Risk-of-bias assessment

The Cochrane risk-of-bias 2 (ROB2) tool was utilized to assess the risk of bias of the included RCTs. A total of five domains were evaluated, which included the randomization process, deviation from intended intervention, incomplete outcome data, measurement of the outcomes, and selective reporting (Sterne et al., 2019). This assessment was conducted independently by two reviewers, with a third researcher resolving any disagreements. Small-study effect (publication bias) was assessed using comparison-adjusted funnel plots (Chaimani & Salanti, 2012).

### Statistical analysis

The outcomes were evaluated utilizing either random-effects or fixed-effects models, depending on heterogeneity. The I^2^ index in the heterogeneity test was utilized to signify literature heterogeneity (Bowden et al., 2011). When the statistical heterogeneity was significant (I^2^ > 50%), the random-effects model was selected. Otherwise, the fixed-effect model was adopted for analysis (Higgins et al., 2003). The results of consistent modeling included the Deviance Information Criterion (DIC), which was further compared with the DIC of inconsistent modeling. If the difference was within 5, it indicated that the data met the premise of consistency. For continuous variables, the mean difference (MD) was utilized to measure the effect size. For binary categorical variables, the odds ratio (OR) was used as the scale. All results were presented as 95% confidence intervals (CIs). A 95% CI was deemed statistically insignificant if it included the value of 0. Publication bias was evaluated using funnel plots. The NMA was conducted using Stata software (V15.0, Stata Corp LLC, College Station, TX, USA) based on the Bayesian model. Data were preprocessed using the network group command, and an evidence network was created for each indicator. The therapeutic properties of the indicators were categorized to calculate the surface under the cumulative ranking curve (SUCRA), and the probability distribution was graphed.

In the evidence network map, the dots symbolized interventions. The size of the dot reflected the number of patients receiving interventions, with larger dots indicating more patients. The line connecting the two dots signified a direct comparison between the two therapies, while the width of the line corresponded to the number of RCTs (Wang et al., 2023). The SUCRA value was depicted as a percentage, where a higher percentage indicated that the intervention had a greater likelihood of being the preferred option, while a value of zero indicated the potential ineffectiveness of the intervention (Uhlmann, Jensen & Kieser, 2018). If a closed loop existed, the node-splitting approach was employed to detect any discrepancies. Funnel plots were employed to evaluate publication bias and the impact of a limited sample size (Page, McKenzie & Higgins, 2018). Ultimately, the literature’s quality was assessed utilizing Review Manager 5.4 software (RevMan 5.4, The Cochrane Collaboration, Oxford, UK). The cell doses were categorized into three groups: low ((0 < cells < 20) ×10^6^), moderate ((20 ≤ cells < 50) ×10^6^), and high (cells ≥ 50 × 10^6^) based on previous dose stratifications in clinical and experimental MSC studies (Huang et al., 2023; Lu et al., 2020; Zhao et al., 2019).

### Evaluation of confidence in findings

The confidence in NMA effect estimates was evaluated using the Confidence In Network Meta-Analysis (CINeMA) framework, which included six domains: within-study bias, across-study bias, indirectness, imprecision, heterogeneity, and incoherence (Higgins, Julian & Green, 2008).

## Results

### Literature screening results

**Figure 1:**
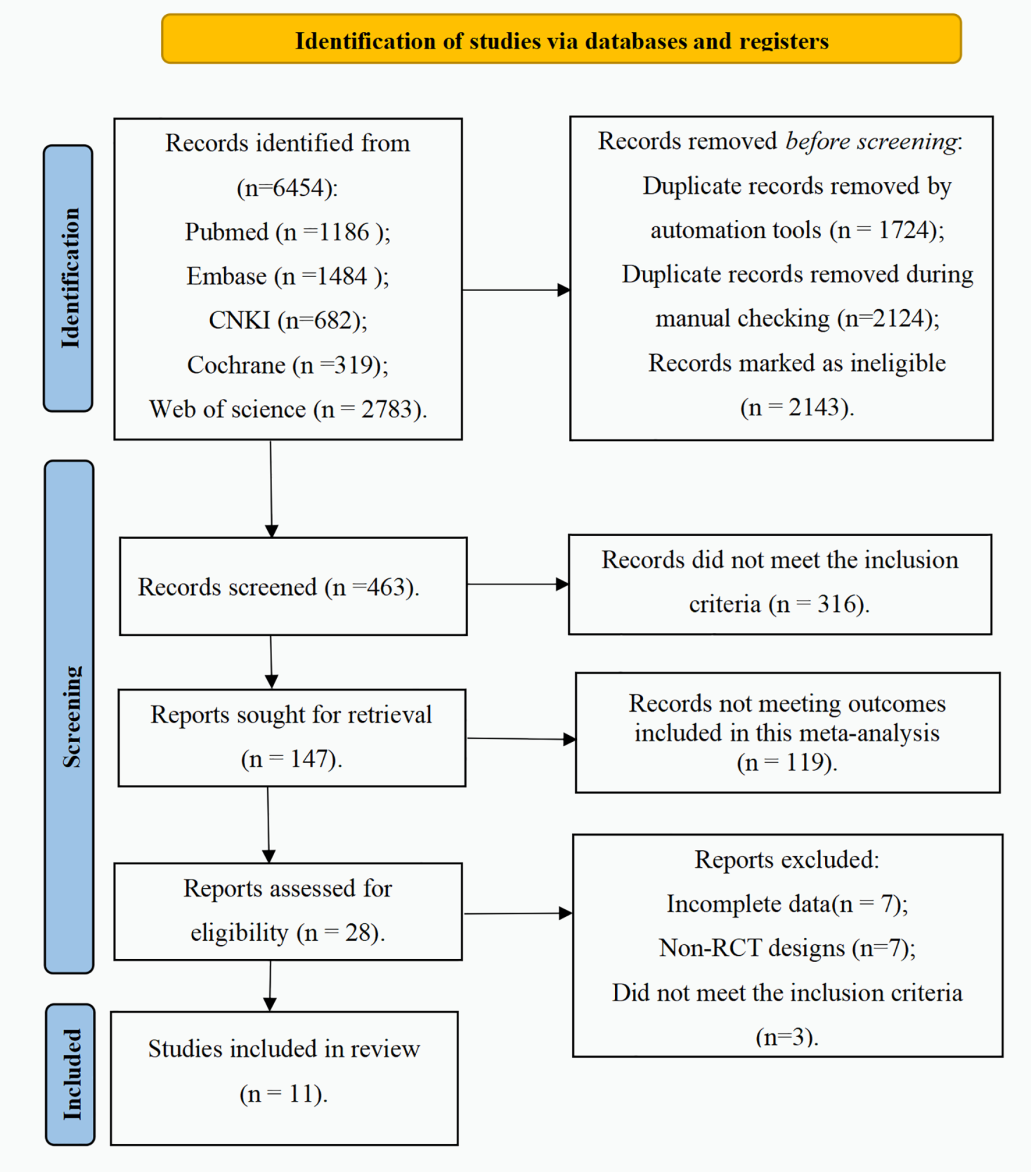
Flowchart of literature screening.

A total of 6,454 records were identified from PubMed, Embase, CNKI, Cochrane Library, and Web of Science (Fig. 1). After 1,724 duplicates were removed using EndNote, 4,730 records remained. During manual checking, 2,124 duplicate records were excluded. Afterward, 2,143 ineligible records were removed, including non-clinical articles, animal-only studies, editorials, case reports, meta-analyses, conference abstracts, pilot studies, and protocols. The titles and abstracts of the remaining 463 records were screened, and 316 were excluded because they did not meet the inclusion criteria (non-KOA population, non-MSC interventions, non-randomized designs, or unrelated outcomes). Among the remaining 147 records, 119 records were excluded for irrelevant outcomes. The full texts of the remaining 28 reports were assessed. Seventeen reports were excluded, of which seven lacked complete data, seven employed non-randomized designs, and three did not meet the predefined inclusion criteria. Additionally, due to ineligible source data and predefined outcomes, no RCTs on synovium-derived MSCs were included. Finally, 11 RCTs were included.

### Characteristics of included RCTs

The characteristics of included RCTs (*n* = 11) are outlined in Table 1. These RCTs involved 635 participants, with 354 in the experiment group and 281 in the control group. The cell dosages varied from 5 * 10^6^ to 100 * 10^6^.

**Table 1:** Characteristics of the included articles in the study.

**Reference**	**Author**	**Country**	**Sample size**	**Gender** **(M/F)**	**Mean age**	**BMI**	**K-L grade**	**Tissue**	**Origin**	**Cell doses**	**Intervention**	**Outcomes**
Zhao et al. (2019)	Zhao, 2019	China	L_ADSCS: 6	5/13	52.05	25.63	II, III	Allogeneic	ADSCS	10*10^6^	IA injection once	F1; F2; F3; F5
			M_ADSCS:6		59.58	23.73				20*10^6^		
			H_ADSCS:6		52.69	24.08				50*10^6^		
Vega et al. (2015)	Vega, 2015	Spain	M_BMSCS:15	11/19	56.60	NR	II, III, IV	Allogeneic	BMSCS	40*10^6^	IA injection once	F1, F4, F7
			Control: 15		57.30				HA			
Sadri et al. (2023)	Sadri, 2023	Iran	H_ADSCS:20	4/36	52.85	29.12	II, III	Allogeneic	ADSCS	100*10^6^	IA injection once	F1, F6, F4
			Control: 20		56.12	28.37			Saline			
Lu et al. (2020)	Lu, 2020	China	L_ADSCS:7	3/19	59.29	27.77	II, III	Allogeneic	ADSCS	10*10^6^	IA injection once	F1, F2, F3, F4
			M_ADSCS:8		57.31	26.69				20*10^6^		
			H_ADSCS:7		57.21	24.51				50*10^6^		
Jo et al. (2017)	Jo, 2017	South Korea	L_ADSCS:3	3/15	61.80	26.00	III, IV	Allogeneic	ADSCS	10*10^6^	IA injection once	F1, F4
			M_ADSCS:3							50*10^6^		
			H_ADSCS:12							100*10^6^		
Kim et al. (2023)	Kim, 2023	South Korea	H_ADSCS:125	65/187	63.70	26.30	III	Autologous	ADSCS	100*10^6^	IA injection once	F1
			Control: 127		63.80	25.90			Saline			
Ho et al. (2022)	Ho, 2022	South Korea	L_BMSCS:10	8/12	56.70	25.4	II, III	Autologous	BMSCS	6*10^6^	IA injection once	F1, F4, F5
			Control:10		59.10	26.00			HA			
Emadedin et al. (2018)	Emadedin, 2018	Iran	M_BMSCS:19	27/16	51.70	30.20	II, III, IV	Autologous	BMSCS	40*10^6^	IA injection once	F1, F4
			Control:24		54.70	31.50			Saline			
Matas et al. (2019)	Matas, 2019	Chile	L_UCSCS:10	13/16	56.10	27.60	II, III	Allogeneic	UCSCS	20*10^6^	IA injection once	F1, F4
			M_UCSCS:10		56.70	27.40				20*10^6^*2	IA injection twice	
			Control: 9		54.80	27.90			HA			
Ha et al. (2018)	Ha, 2019	China	L_UCSCS:43	23/63	57.00	25.80	II, III	Allogeneic	UCSCS	5*10^6^*2	IA injection twice	F4, F5
			Control:43		56.20	24.90			HA			
Yang et al. (2017)	Yang, 2017	China	L_UCSCS:15	11/33	70.60	24.60	II, III	Allogeneic	UCSCS	15*10^6^*2	IA injection twiceIA injection fourth	F4
			M_UCSCS:13		71.50	27.10				15*10^6^*4		
			Control:16		72.20	25.60			HA			

**Notes.**

Abbreviations ADSCS Adipose-Derived Mesenchymal Stem Cells BMSCS Bone Marrow-Derived Mesenchymal Stem Cells UCSCS Umbilical Cord-Derived Mesenchymal Stem Cells IA Intra-articular L_ Low Dose M_ Moderate Dose H_ High Dose m month y year WOMAC Western Ontario and McMaster Universities Arthritis Index total score VAS Visual Analog Scale AE Adverse Event HA Hyaluronic Acid F1 WOMAC F2 SF_36 F3 WORMS F4 VAS F5 T2 values F6 KOOS F7 LEQUESNE

### Risk of bias assessment

**Figure 2:**
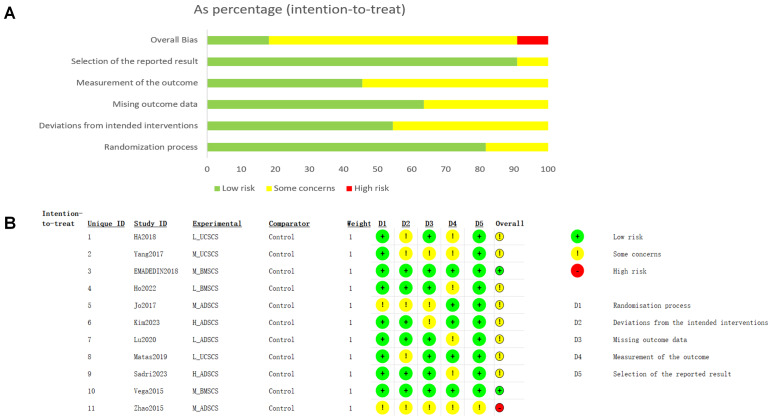
Risk of bias assessment diagram.

Eleven RCTs were assessed and did not show any fabricated or incomplete data. The risk of incomplete reporting and early discontinuation was graded as low. No other biases were identified or discussed in any RCTs. The risk of bias is shown in Fig. 2.

### Outcomes

The primary outcomes included long-term (12 months) WOMAC scores, long-term VAS scores, and AEs. Medium-term (6 months) VAS, medium-term (6 months) WOMAC, and short-term (3 months) VAS scores were secondary outcomes. The short-term (3 months) WOMAC scores were not analyzed due to insufficient data from the included studies.

#### Primary Outcomes

##### Long-term WOMAC (12 months).

**Figure 3:**
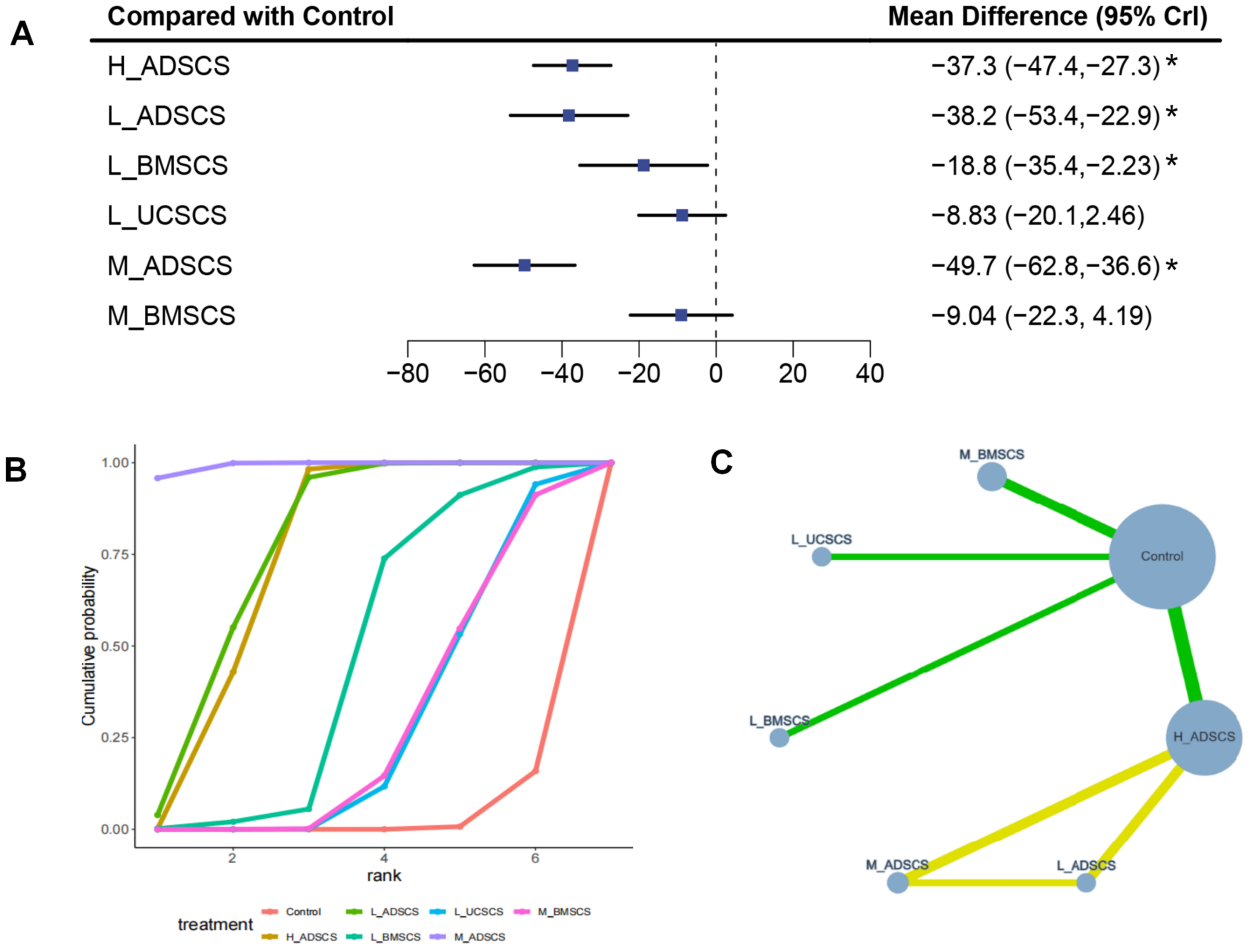
Meta-analysis of treatment effects of long-term (12 months) WOMAC total scores. (A) Forest plot of WOMAC after 12 months; (B) Cumulative probability graph of WOMAC after 12 months; (C) Network diagram of WOMAC after 12 months. Abbreviations: ADSCS, Adipose-Derived Mesenchymal Stem Cells; BMSCS, Bone Marrow-Derived Mesenchymal Stem Cells; UCSCS, Umbilical Cord-Derived Mesenchymal Stem Cells; L_, Low Dose; M_, Middle Dose; H_, High Dose; *: statistical significance.

**Figure 4:**
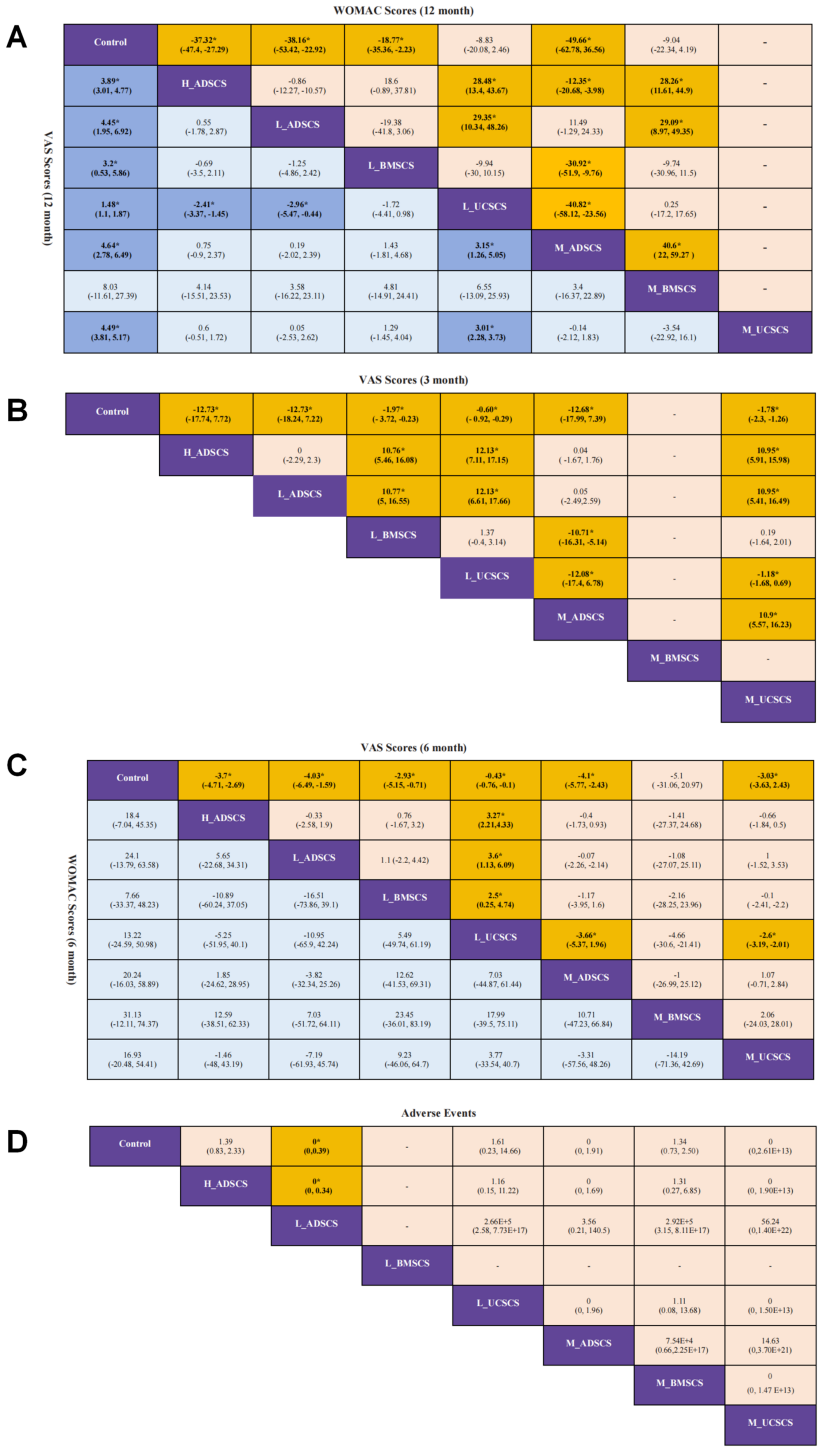
League table of outcome analysis. (A) League table of WOMAC total Scores (12 months) and VAS Scores (12 months); (B) League table of VAS Scores (3 months); (C) League table of WOMAC total Scores (6 months) and VAS Scores (6 months); (D) League table of adverse events. The values in each cell show the relative effectiveness and 95% CI of the treatment on the column, compared to the treatment on the row. For both WOMAC and VAS, if the 95% CI is negative, the effectiveness of the intervention on the column is significantly better than that on the row; if the 95% CI is positive, the effectiveness of the intervention on the row is significantly better than that on the column. For AEs with binary variables, a 95% CI of (0,1) indicates that the incidence of AEs of the intervention on the row is significantly lower than that on the column. *: statistical significance.

Six studies, with 160 individuals and six interventions, provided long-term post-treatment WOMAC scores. Moderate-dose adipose derived MSCs (M_ADSCS) demonstrated the most consistent and robust long-term improvement in WOMAC scores, whereas low-dose UCSCS (L_UCSCS) and the control showed the weakest performance. The evidence network mostly focused on Control, with low-dose (L_ADSCS), M_ADSCS, and high-dose ADSCS (H_ADSCS) forming a closed loop (Fig. 3C). Due to the limited number of investigations, local discrepancies cannot be compared. H_ADSCS (MD = −37.3, 95% CI [−47.4 to −27.3]), L_ADSCS (MD = −38.2, 95% CI [−53.4 to −22.9]), low-dose BMSCS (L_BMSCS) (MD = −18.8, 95% CI [−35.4 to −2.23]), and M_ADSCS (MD = −49.7, 95% CI [−62.8 to −36.6]) exhibited superior effects on the long-term WOMAC score (*P* < 0.05) to the Control (Fig. 3A). H_ADSCS (MD = −28.48, 95% CI [−43.67 to −13.4]) and L_ADSCS (MD = −29.35, 95% CI [−48.26 to −10.34]) greatly diminished the long-term WOMAC score compared with L_UCSCS. H_ADSCS (MD = −28.26, 95% CI [−44.9 to −11.61]), M_ADSCS (MD = −40.6, 95% CI [−59.27 to −22]), and L_ADSCS (MD = −29.09, 95% CI [−49.35 to −8.97]) notably diminished the long-term WOMAC score compared with moderate-dose BMSCS (M_BMSCS) (*P* < 0.05) (Fig. 4A). The SUCRA RANK revealed that M_ADSCS (SUCRA: 99.3%), L_ADSCS (SUCRA: 75.8%), and H_ADSCS (SUCRA: 73.5%) were the optimal therapies for long-term WOMAC score. L_UCSCS (SUCRA: 26.5%) and Control (SUCRA: 2.7%) had the lowest effectiveness (Fig. 3B and Table S1). Seven two-by-two comparisons were made, with no notable heterogeneity (min *I*^2^ = 20.2%, max *I*^2^ = 64.2%). No marked difference was observed in any pairwise comparisons (Table S2).

### Long-term VAS (12 months)

**Figure 5:**
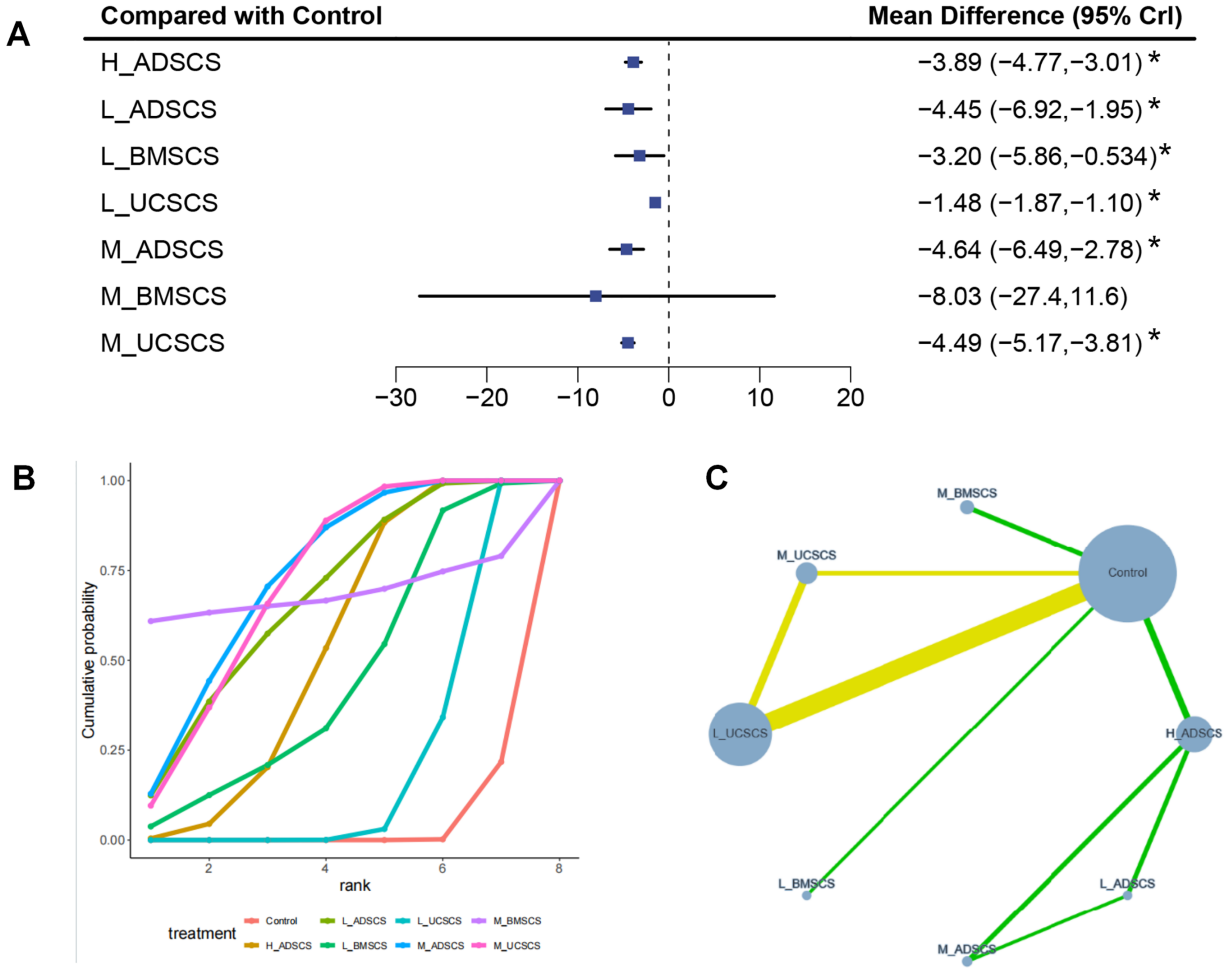
Meta-analysis of treatment effects of long-term (12 months) VAS score. (A) Forest plot of VAS after 12 months; (B) Cumulative probability graph of VAS after 12 months; (C) Network diagram of VAS after 12 months. *: statistical significance.

Nine RCTs, with 292 individuals and seven interventions, provided long-term post-treatment VAS scores. M_ADSCS exhibited the greatest long-term pain-relief probability, while L_UCSCS and the control remained the least effective. All interventions, except M_BMSCS, significantly reduced long-term VAS scores. The evidence network mostly focused on Control, with L_ADSCS, M_ADSCS, and H_ADSCS forming a closed loop (also happened among Control, L_UCSCS, and moderate-dose UCSCS (M_UCSCS)) (Fig. 5C). Then the node-splitting method confirmed good consistency, with no heterogeneity (*P* > 0.05) (Fig. S1). Compared to Control, all interventions, except M_BMSCS, had favorable effects (Fig. 5A), including H_ADSCS (MD = −3.89, 95% CI [−4.77 to −3.01]), L_ADSCS (MD = −4.45, 95% CI [−6.92 to −1.95]), L_BMSCS (MD = −3.20, 95% CI [−5.86 to −0.534]), L_UCSCS (MD = −1.48, 95% CI [−1.87 to −1.10]), M_ADSCS (MD = −4.64, 95% CI [−6.49 to −2.78]), and M_UCSCS (MD = −4.49, 95% CI [−5.17 to −3.81]). Additionally, H_ADSCS (MD = −2.41, 95% CI [−3.37 to −1.45]) and L_ADSCS (MD = −2.96, 95% CI [−5.47 to −0.44]) considerably reduced the long-term VAS score compared with L_UCSCS (*P* < 0.05) (Fig. 5A). The SUCRA RANK revealed that M_ADSCS (SUCRA: 73.0%), M_UCSCS (71.4%), and M_BMSCS (SUCRA: 68.5%) were the optimal therapies for long-term WOMAC scores. L_UCSCS (SUCRA: 19.6%) and Control (SUCRA: 3.1%) had the lowest effectiveness (Fig. 5B and Table S1). Eight two-by-two comparisons were made, with no substantial heterogeneity (min *I*^2^ = 0.0%, max *I*^2^ = 56.1%). No marked difference was observed in any pairwise comparisons (Table S2).

#### Secondary outcomes

##### Medium-term WOMAC (6 months).

**Figure 6:**
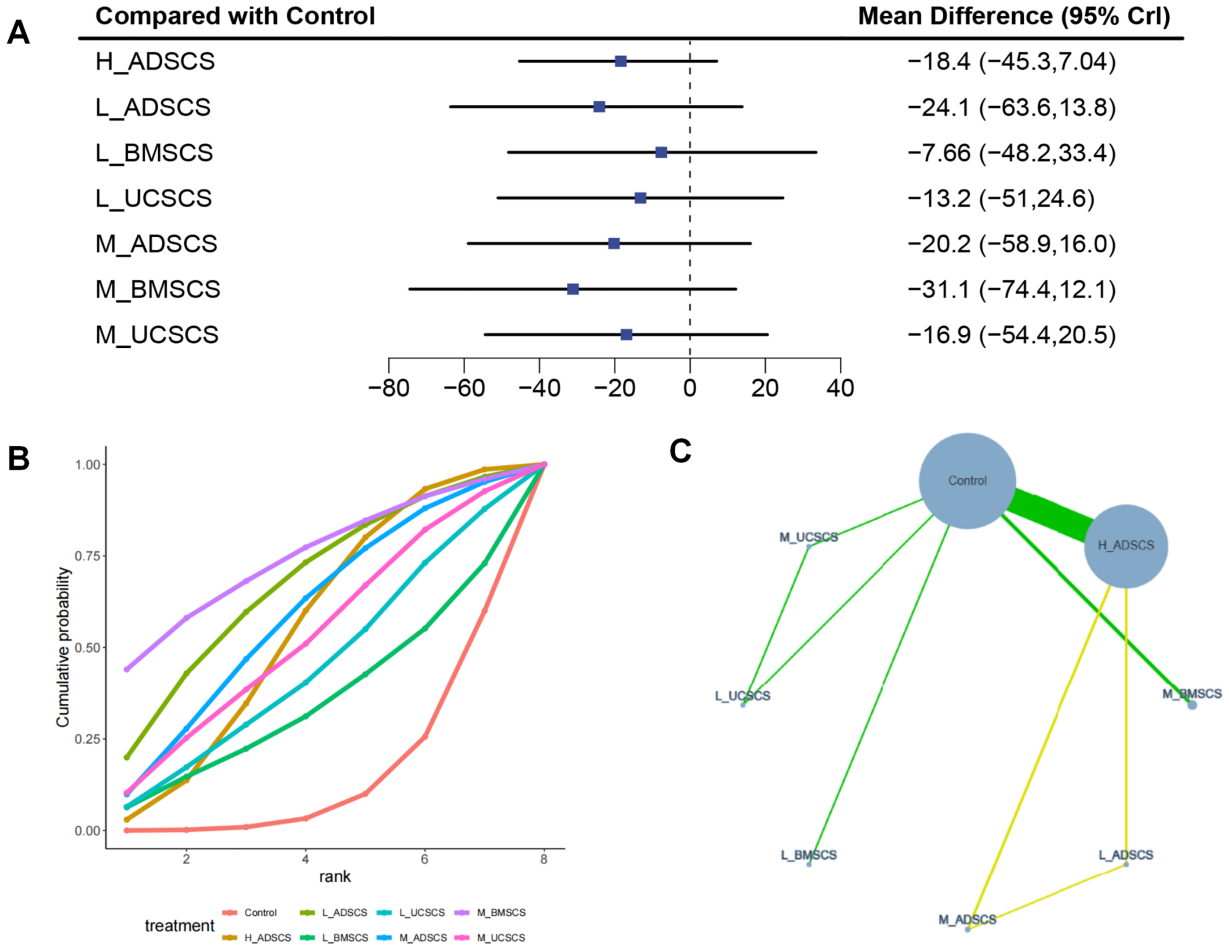
Meta-analysis of treatment effects of medium-term (6 months) WOMAC total scores. (A) Forest plot of WOMAC after 6 months; (B) Cumulative probability graph of WOMAC after 6 months; (C) Network diagram of WOMAC after 6 months.

Seven studies, with 424 individuals and seven interventions, provided medium-term post-treatment WOMAC scores. No MSC intervention showed a statistically significant advantage over control or other treatments for WOMAC scores, although M_BMSCS ranked the highest in SUCRA value. The evidence network mostly focused on Control, with L_ADSCS, M_ADSCS, and H_ADSCS forming a closed loop (Fig. 6C). However, due to the limited number of investigations, local discrepancies were not compared. None therapies had a superior impact on the medium-term WOMAC score to the control group (Fig. 6A). Moreover, there was no statistical significance among interventions (Fig. 4C). The SUCRA RANK revealed that M_BMSCS (SUCRA: 74.2%) were the most effective therapy for medium-term WOMAC scores. Control (SUCRA: 14.3%) had the lowest effectiveness (Fig. 6B and Table S1). Nine two-by-two comparisons were made, revealing marked heterogeneity (min I^2^ = 0%, max *I*^2^ = 96.4%). No considerable difference was observed in any pairwise comparisons (Table S2).

##### Short-term VAS (3 months).

**Figure 7:**
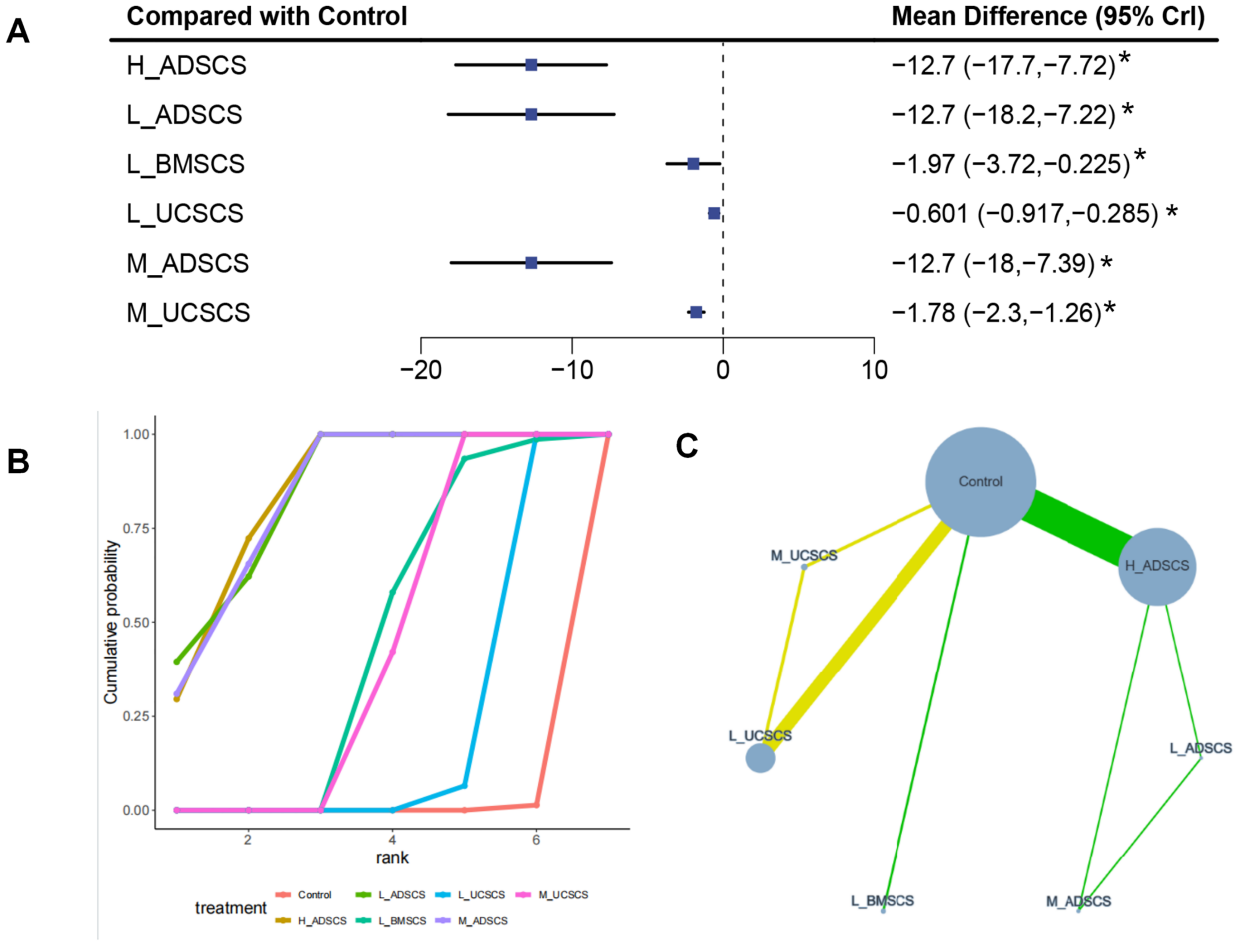
Meta-analysis of treatment effects of short-term (3 months) VAS score. (A) Forest plot of VAS after 3 months; (B) Cumulative probability graph of VAS after 3 months; (C) Network diagram of VAS after 3 months. *: statistical significance.

Six studies, with 464 individuals and six interventions, provided short-term VAS scores. All MSC interventions reduced VAS scores more than control, with H_ADSCS ranking best and ADSCS providing greater short-term pain relief than UCSCS. The evidence network mostly focused on Control, with L_ADSCS, M_ADSCS, and H_ADSCS forming a closed loop, and Control, L_UCSCS, and M_UCSCS forming a closed loop (Fig. 7C). Due to the limited number of investigations, local discrepancies were not compared. All interventions demonstrated superior effects on long-term WOMAC score to Control (*P* < 0.05) (Fig. 7A). These interventions covered H_ADSCS (MD = −12.7, 95% CI [−17.7 to −7.72]), L_ADSCS (MD = −12.7, 95% CI ([−18.2 to −7.22]), L_BMSCS (MD = −1.97, 95% CI [−3.72 to −0.225]), L_UCSCS (MD = −0.601, 95% CI [−0.917 to −0.285]), M_ADSCS (MD = −12.7, 95% CI [−18 to −7.39]), and M_UCSCS (MD = −1.78, 95% CI [−2.3 to −1.26]). Furthermore, H_ADSCS (MD =−12.13, 95% CI [−17.15 to −7.11]) and L_ADSCS (MD = −12.13, 95% CI [−17.66 to −6.61]) notably reduced the short-term VAS score compared with L_UCSCS. H_ADSCS (MD = −10.95, 95% CI [−15.98 to −5.91]), M_ADSCS (MD = −10.9, 95% CI [−16.23 to −5.57]), and L_ADSCS (MD = −10.95, 95% CI [−16.49 to −5.41]) greatly lowered the short-term VAS score compared with M_UCSCS (*P* < 0.05) (Fig. 4B). The SUCRA RANK revealed that H_ADSCS (SUCRA: 83.6%) was the optimal therapy for long-term WOMAC score. Control (SUCRA: 0.2%) had the lowest effectiveness (Fig. 7B and Table S1). Eight two-by-two comparisons were made, revealing pronounced heterogeneity (min *I*^2^ = 0.0%, max *I*^2^ = 81.0%). No marked difference was observed in any pairwise comparisons (Table S2).

##### Medium-term VAS (6 months).

**Figure 8:**
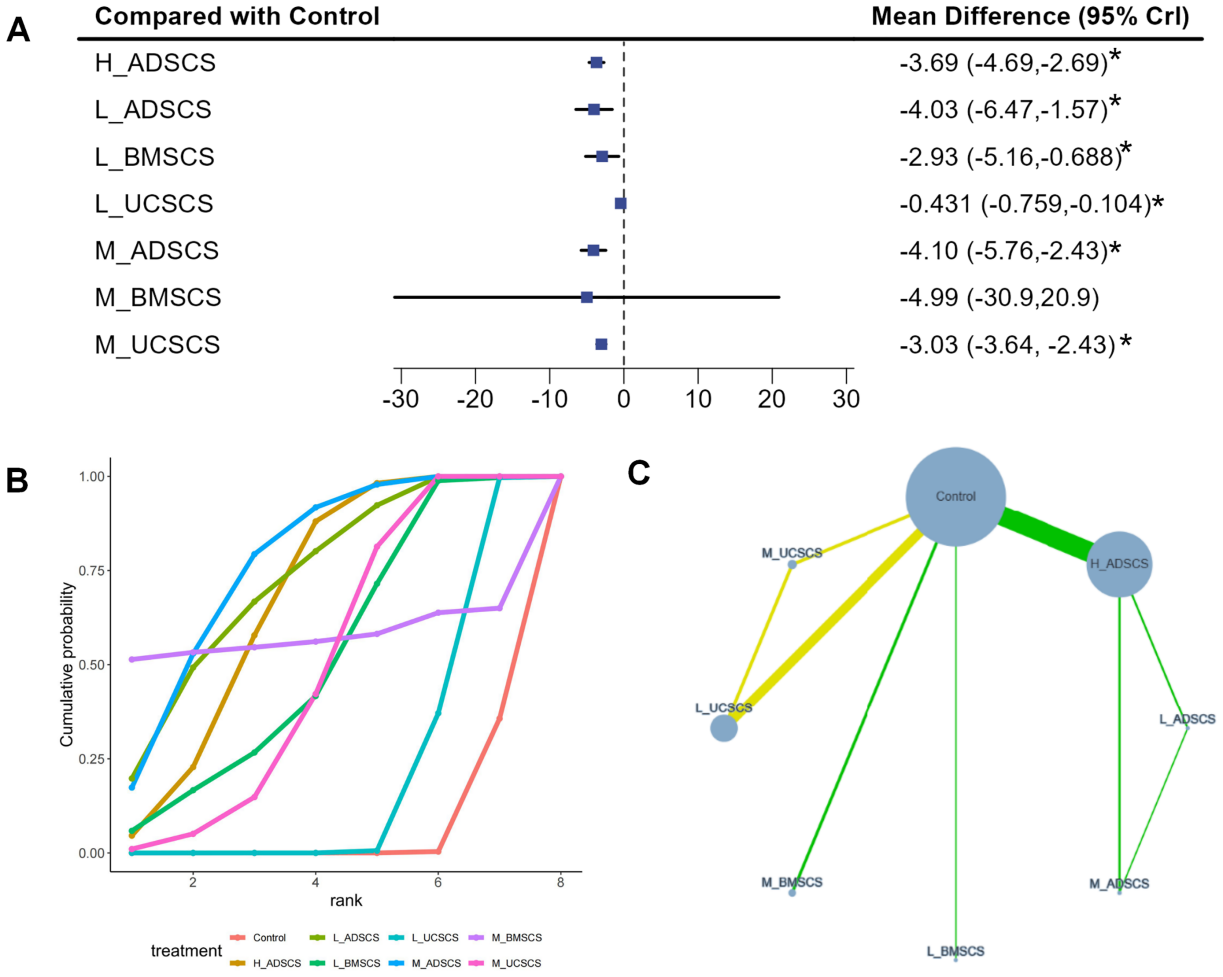
Meta-analysis of treatment effects of medium-term (6 months) VAS score. (A) Forest plot of VAS after 6 months; (B) Cumulative probability graph of VAS after 6 months; (C) Network diagram of VAS after 6 months. *: statistical significance.

Nine studies, with 554 individuals and seven interventions, provided medium-term post-treatment VAS scores. In these trials, most MSC treatments (all except M_BMSCS) reduced VAS scores more than the control. M_ADSCS ranked highest while M_BMSCS did not demonstrate superiority over the control group. The evidence network mostly focused on Control, with L_ADSCS, M_ADSCS, and H_ADSCS forming a closed loop (also happened among Control, L_UCSCS, and M_UCSCS) (Fig. 8C). Due to the limited number of investigations, local discrepancies were not compared. Compared to Control, all interventions, except M_BMSCS, exhibited favorable effects on the medium-term VAS score (*P* < 0.05) (Fig. 8A), including H_ADSCS (MD = −3.69, 95% CI [−4.69 to −2.69]), L_ADSCS (MD = −4.03, 95% CI [−6.47 to −1.57]), L_BMSCS (MD = −2.93, 95% CI [−5.16 to −0.688]), L_UCSCS (MD = −0.431, 95% CI [−0.759 to −0.104]), M_ADSCS (MD = −4.1, 95% CI [−5.76 to −2.43]), M_BMSCS (MD = −4.99, 95% CI [−30.9 to −20.9]), and M_UCSCS (MD = −3.03, 95% CI [−3.64 to −2.43]). Additionally, L_ADSCS (MD = −3.6, 95% CI [−6.09 to −1.13]) and L_BMSCS (MD = −2.5, 95% CI [−4.74 to −0.25]) notably reduced the medium-term VAS score compared with L_UCSCS (*P* < 0.05) (Fig. 4C). The SUCRA RANK revealed that M_ADSCS (SUCRA: 77.0%) was the most effective therapy for medium-term VAS scores. Control (SUCRA: 5.2%) had the lowest effectiveness (Fig. 8B and Table S1). Nine two-by-two comparisons were made, revealing pronounced heterogeneity (min *I*^2^ = 0.0%, max *I*^2^ = 99.7%). No marked difference was observed in any pairwise comparisons (Table S2).

##### AEs.

**Figure 9:**
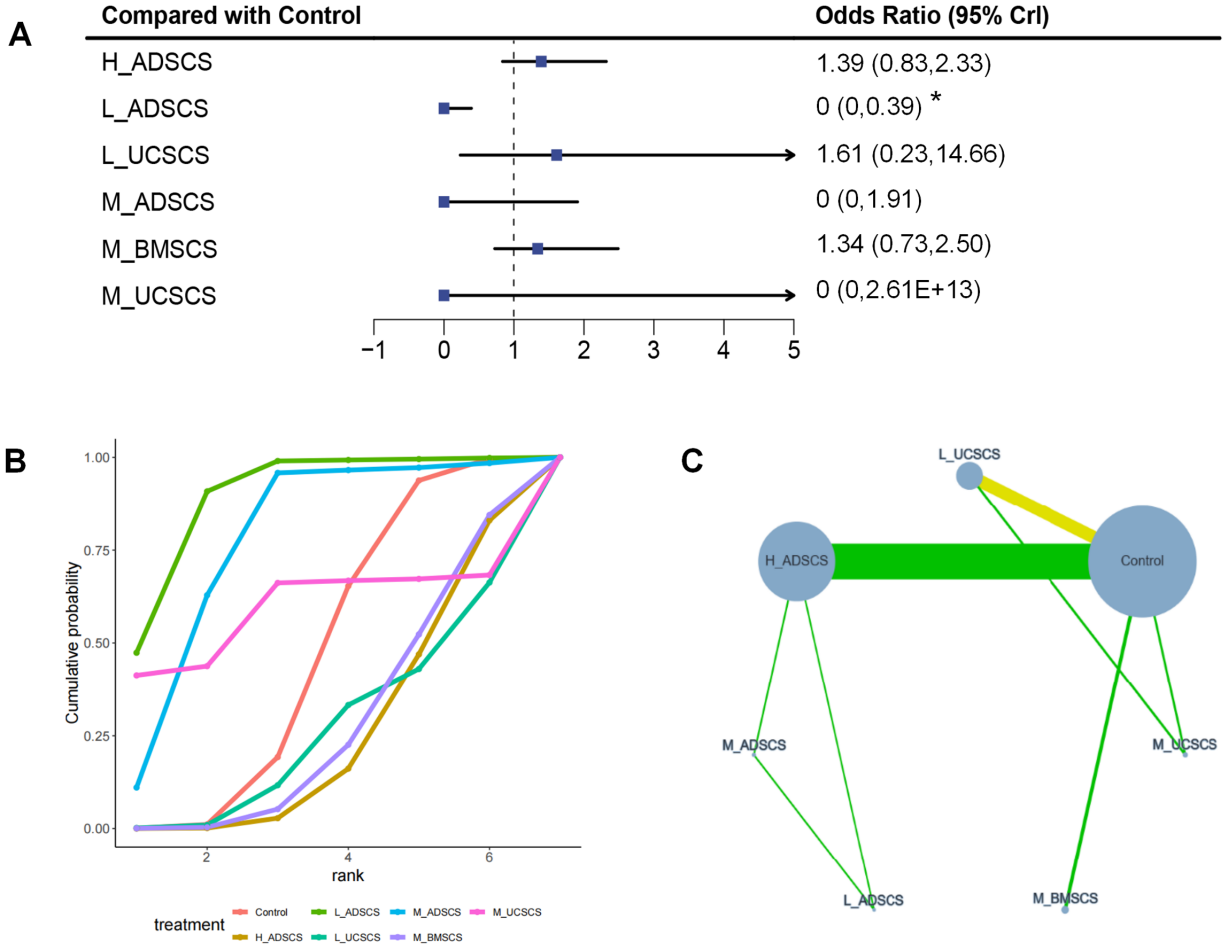
Meta-analysis of treatment effects of Adverse events (AEs). (A) Forest plot of AEs; (B) Cumulative probability graph of AEs; (C) Network diagram of AEs. *: statistical significance.

Six RCTs, with 464 individuals and six interventions, provided data on AEs. Most MSC treatments yielded similar AE rates to the control. L_ADSCS showed significantly fewer AEs than both control and H_ADSCS and ranked as the safest option, while H_ADSCS was most likely to induce AEs. The evidence network mostly focused on Control, with L_ADSCS, M_ADSCS, and H_ADSCS forming a closed loop (also happened among Control, L_UCSCS, and M_UCSCS) (Fig. 9C). Due to the limited number of investigations, local discrepancies were not compared. Compared to Control, no therapies had a superior impact on AEs, except L_ADSCS (OR=2.12E-06, 95% CI [3.28E−18 to 0.393]), exhibiting a lower rate of AEs (*P* < 0.05) (Fig. 9A). The SUCRA RANK revealed that L_ADSCS (SUCRA: 85.9%) may be the intervention with the lowest likelihood of AEs. H_ADSCS (SUCRA: 23%) was most likely to cause AEs (Fig. 9B and Table S1). Eight two-by-two comparisons were made, revealing no marked heterogeneity (min *I*^2^ = 0.0%, max *I*^2^ = 56.1%). No remarkable difference was observed in any pairwise comparisons (Table S2). Moreover, the incidence of AEs of L_ADSCS injection was significantly lower than that of H_ADSCS (OR = 0, 95% CI [0–0.34]) (Fig. 4D).

### Risk-of-bias assessment

According to the ROB2 assessment, two RCTs were judged to have a low risk of bias across all domains, one study had an overall high risk for all five domains, and nine studies had some concerns, particularly in the domain of deviations from intended interventions, due to a lack of blinding (Fig. 2B). In terms of CINeMA, some concerns were identified related to imprecision, heterogeneity, and incoherence. Consequently, the overall confidence in most comparisons was rated as moderate, except that Control *vs* H_ADSCS was rated as high in long-term VAS (Supplementary Appendix 2). Outcomes were tested for publication bias in funnel plots (Fig. S2). The funnel plot of VAS (12 months) was asymmetric, denoting a publication bias or small sample effect, which may influence the results. The funnel plots for other indicators were symmetrical, implying a low possibility of publication bias.

## Discussion

A total of 11 RCTs were enrolled, with 602 KOA subjects from 19 experiment groups. Bayesian NMA compared the short-term, medium-term, and long-term efficacy of MSCs from different tissue sources (ADSCS, BMSCS, UCSCS) and at different doses (low, medium, high). The results revealed that compared with the control group, all interventions, except M_BMSCS, could effectively improve the pain score (VAS). H_ADSCS had the more appropriate short-term analgesic effect, while M_ADSCS had the more appropriate medium-term and long-term analgesic effect. Moreover, all interventions had no pronounced medium-term effect on WOMAC, while in terms of long-term effect, H_ADSCS, M_ADSCS, L_ADSCS, and L_BMSCS could considerably improve WOMAC, and M_ADSCS was optimal.

MSCs are pluripotent stem cells with multidirectional differentiation, immunoregulation, and low immunogenicity (Kyurkchiev et al., 2014). Compared with steroids, hyaluronic acid, and platelet-rich plasma, MSCs show promising potential in promoting cartilage regeneration and improving joint microenvironment (Wang et al., 2015). Consistent with previous studies, our results showed the superiority of ADSCS to MSCs from other tissue sources. For example, Chen et al. (2024) and Wang et al. (2015) found that ADSCS were more effective than BMSCS and UCSCS in pain relief. From a mechanistic perspective, *in vitro* studies have shown more favorable immunomodulatory profiles of ADSCS than BMSCS and UCSCS, including stronger suppression of activated immune cells and higher secretion of key immunoregulatory cytokines, such as IL-6 and TGF-β1 (Melief et al., 2013; Ribeiro et al., 2013). In osteoarthritis (OA)-related models, ADSCS and their secretomes or small extracellular vesicles reduce inflammatory and catabolic mediators and support cartilage extracellular matrix synthesis in chondrocytes and synoviocytes from OA patients, thereby creating a chondroprotective, pro-regenerative milieu (Cavallo et al., 2021; Manferdini et al., 2013). Collectively, these findings support that ADSCS provide a particularly favorable biological profile for immunoregulation and cartilage protection, which is consistent with the present analysis.

Direct comparisons of the efficacy of MSCs from different doses at different tissue sources are lacking, although studies have tried to make indirect comparisons through meta-analyses. For example, Huang et al. (2023) included 16 RCTs to analyze the clinical efficacy of ADSCS at different doses. They showed that H_ADSCS had a more significant effect on improving joint function and relieving pain than low and medium doses of ADSCS. Muthu et al. (2021) reported that BMSCS of 5–10 × 10^7^ cells introduced significant functional improvements compared to BMSCS of <1 × 10^7^ cells, 1–5 ×10^7^ cells, and >10 × 10^7^ cells. However, these studies have mostly focused on tissue-specific MSCs, lacking comprehensive comparisons of the efficacy between different sources and different doses. Jo et al. (2017) observed that H_ADSCS (100*10^6^) led to significant improvements in the short and medium term, while L_ADSCS and M_ADSCS did not demonstrate notable improvement. This discrepancy may be attributed to the differences in patients’ initial disease conditions. They included 18 patients, with six patients at the K-L stage IV and 12 at the K-L stage III. In contrast, the patients included in our study were mainly at the K-L stage II and III. Therefore, MSC doses may also depend on KOA severity. Meanwhile, Muthu et al. (2025) demonstrated the significant improvement in pain relief and functional scores of KOA patients who received injections of bone marrow aspirate concentrate with normal BMI (*n* = 43) compared to obese (*n* = 15) and overweight patients (*n* = 10). Therefore, obesity, comorbidity, or age also should be considered when choosing the dose. In addition, the difference in efficacy between autologous and allogeneic sources of MSCs is also worth noting, although subgroup analysis of these two sources could not be performed in this paper due to the lack of data. Previous clinical trials of intra-articular autologous MSC injections for KOA and other large-joint OA have reported good long-term safety, with no evidence of immune-mediated rejection (Emadedin et al., 2015). In conclusion, this study found that M-ADSCS may yield the greatest improvement in KOA treatment. In clinical practice, MSCs need to be selected after comprehensive analysis of patients’ conditions.

For joint function (WOMAC), our results revealed that MSC injection did not show significant medium-term efficacy compared with the control interventions, while most interventions showed significant long-term efficacy, which was consistent with previous findings (Lamo-Espinosa et al., 2016; Lu et al., 2019). WOMAC total score in the experiment groups at 6 months was not notably different from that in the control group. However, noticeable improvements were observed in the total WOMAC score at 12 months. From the perspective of cell biology, MSCs have high proliferation and differentiation potential (Caplan, 2007), which, however, requires a certain period. Consistent with this time-dependent effect, our results indicated that M_ADSCS produced greater long-term improvements in joint function than the other interventions. It is possibly related to the pronounced anti-inflammatory, immunomodulatory, and anti-apoptotic properties of ADSCS. A moderate dose is also more likely to provide a stable intra-articular environment for matrix synthesis. In contrast, high doses may impair cell survival and function because of cell overcrowding and competition for limited nutrients and oxygen, thereby hindering cell differentiation and matrix synthesis. This is supported by our analysis of AEs, which showed a higher incidence of AEs in the H_ADSCS group than in the other groups, although no serious AEs were reported.

For pain (VAS), our results demonstrated that MSCs in all groups could significantly improve pain symptoms at all periods, except M_BMSCS. In addition, H_ADSCS had better short-term analgesic effects based on the SUCRA ranking, while M_ADSCS had better long-term analgesic effects. Similarly, Gupta et al. (2016) found that low, medium, and high doses of MSCs showed significant improvements in VAS, and the VAS score was greatly decreased after intervention with medium-dose MSCs (25  × 10^6^ cells) compared to that of low and high doses at 12 months. Although the specific mechanism of MSCs in relieving pain remains uncertain, MSC-mediated improvements in KOA are increasingly attributed to their secretory activity. MSCs can release anti-inflammatory cytokines, growth factors, and extracellular vesicles that modulate the joint environment and mitigate inflammation (Ho et al., 2022). For example, one clinical study reported that MSCs significantly reduced the levels of pro-inflammatory mediators (TNF-α, IL-6) at 6–12 months post-injection and yielded better outcomes than debridement controls (Li et al., 2020). Notably, clinical data on MSC-derived exosomes, which are a key component of MSC secretomes, similarly demonstrated decreased joint inflammation and signs of cartilage repair in OA patients (Wang et al., 2025). Collectively, these findings support that MSCs primarily act *via* paracrine immunomodulation and trophic stimulation of local tissues, which explains the medium-term symptomatic relief observed in KOA after MSC therapy even before structural changes become apparent.

However, over time, higher doses of MSC injection may trigger rapid cell death and apoptosis due to the harsh local microenvironment and intercellular competition, thus affecting their actual survival rate. After apoptosis and lysis, cell debris-released damage-associated molecular patterns may initiate local inflammatory responses, further exacerbating inflammation and pain symptoms (Sengprasert et al., 2023). In contrast, M_ADSCS provided a modest immunoregulatory effect. In addition, extreme changes in the microenvironment caused by H_ADSCS in the short term may stimulate macrophages to polarize into M1 type (pro-inflammatory response), while M_ADSCS may provide more stable and durable effects on improving the microenvironment and then activate macrophages towards M2 polarization (anti-inflammatory/immunosuppressive response) (Saether et al., 2014).

This is the first systematic review and NMA on the efficacy of MSCs from different tissue sources and at different doses. Bayesian NMA addresses the limitations inherent in traditional meta-analysis, which cannot simultaneously evaluate multiple interventions and compare the efficacy of different interventions at different durations. However, this paper also has some limitations. Firstly, although all comparator arms used only two standard intra-articular treatments (hyaluronic acid or normal saline), we cannot completely exclude the possibility that differences between these control modalities contributed to residual heterogeneity. Secondly, the limited sample size may reduce statistical power and affect the generalizability of the results. Furthermore, based on previous studies, the dose analysis in this study was categorized into three levels (low, medium, high); however, such broad grouping may dilute potential differences. Therefore, the findings reflect trends across a wide dosage range rather than identifying a definitive optimal cell count. Thirdly, insufficient data prevent comparisons on the frequency of injections. Fourth, the observed high heterogeneity (I^2^ > 75%) suggests differences in study populations and methodologies. Differences in patient age, OA severity, MSC preparation protocols, and outcome assessment time points may account for this inconsistency. To address these issues, future studies should obtain more available data, standardize MSC preparation protocols, and validate the results through larger-scale, multicenter RCTs. Although the evidence base is limited, this NMA provides structured comparisons of the available studies, highlighting the trend toward the clinical use of M_ADSCS.

## Conclusions

In conclusion, our study found that M_ADSCS may provide greater symptomatic relief for KOA compared with MSCs at other doses from other sources owing to its superiority in pain relief and function improvement. Due to the limitations of this study, more large-sample RCTs are needed to illustrate the efficacy and safety of MSCs from different tissue sources and at different doses.

##  Supplemental Information

10.7717/peerj.20776/supp-1 Supplemental Information 1 PRISMA checklist

10.7717/peerj.20776/supp-2 Supplemental Information 2 Raw data of short-term VAS (3 months) Abbreviation: std.dev: standard deviation; ADSCS: Adipose-Derived Mesenchymal Stem Cells; BMSCS: Bone Marrow-Derived Mesenchymal Stem Cells; UCSCS: Umbilical Cord-Derived Mesenchymal Stem Cells; L_: Low Dose; M_: Middle Dose; H_: High Dose; L_ADSCS: Low-dose Adipose-derived MSCs; M_ADSCS: Medium-dose Adipose-derived MSCs; H_ADSCS: High-dose Adipose-derived MSCs; L_BMSCS: Low-dose Bone Marrow-derived Mesenchymal Stem Cells.

10.7717/peerj.20776/supp-3 Supplemental Information 3 Raw data of medium-term VAS (6 months) Abbreviation: std.dev: standard deviation; ADSCS: Adipose-Derived Mesenchymal Stem Cells; BMSCS: Bone Marrow-Derived Mesenchymal Stem Cells; UCSCS: Umbilical Cord-Derived Mesenchymal Stem Cells; L_: Low Dose; M_: Middle Dose; H_: High Dose; L_ADSCS: Low-dose Adipose-derived MSCs; M_ADSCS: Medium-dose Adipose-derived MSCs; H_ADSCS: High-dose Adipose-derived MSCs; L_BMSCS: Low-dose Bone Marrow-derived Mesenchymal Stem Cells.

10.7717/peerj.20776/supp-4 Supplemental Information 4 Raw data of long-term VAS (12 months) Abbreviation: std.dev: standard deviation; ADSCS: Adipose-Derived Mesenchymal Stem Cells; BMSCS: Bone Marrow-Derived Mesenchymal Stem Cells; UCSCS: Umbilical Cord-Derived Mesenchymal Stem Cells; L_: Low Dose; M_: Middle Dose; H_: High Dose; L_ADSCS: Low-dose Adipose-derived MSCs; M_ADSCS: Medium-dose Adipose-derived MSCs; H_ADSCS: High-dose Adipose-derived MSCs; L_BMSCS: Low-dose Bone Marrow-derived Mesenchymal Stem Cells.

10.7717/peerj.20776/supp-5 Supplemental Information 5 Raw data of medium-term WOMAC (6 months) Abbreviation: std.dev: standard deviation; ADSCS: Adipose-Derived Mesenchymal Stem Cells; BMSCS: Bone Marrow-Derived Mesenchymal Stem Cells; UCSCS: Umbilical Cord-Derived Mesenchymal Stem Cells; L_: Low Dose; M_: Middle Dose; H_: High Dose; L_ADSCS: Low-dose Adipose-derived MSCs; M_ADSCS: Medium-dose Adipose-derived MSCs; H_ADSCS: High-dose Adipose-derived MSCs; L_BMSCS: Low-dose Bone Marrow-derived Mesenchymal Stem Cells.

10.7717/peerj.20776/supp-6 Supplemental Information 6 Raw data of long-term WOMAC (12 months) Abbreviation: std.dev: standard deviation; ADSCS: Adipose-Derived Mesenchymal Stem Cells; BMSCS: Bone Marrow-Derived Mesenchymal Stem Cells; UCSCS: Umbilical Cord-Derived Mesenchymal Stem Cells; L_: Low Dose; M_: Middle Dose; H_: High Dose; L_ADSCS: Low-dose Adipose-derived MSCs; M_ADSCS: Medium-dose Adipose-derived MSCs; H_ADSCS: High-dose Adipose-derived MSCs; L_BMSCS: Low-dose Bone Marrow-derived Mesenchymal Stem Cells.

10.7717/peerj.20776/supp-7 Supplemental Information 7 Raw data of Adverse Events

10.7717/peerj.20776/supp-8 Supplemental Information 8 Supplemental Material

## List of abbreviations

ADSCS: Adipose-Derived Mesenchymal Stem Cells
BMSCS: Bone Marrow-Derived Mesenchymal Stem Cells
UCSCS: Umbilical Cord-Derived Mesenchymal Stem Cells
IA: Intra-articular
L_: Low Dose
M_: Middle Dose
H_: High Dose
L_ADSCS: Low-dose Adipose-derived MSCs
M_ADSCS: Medium-dose Adipose-derived MSCs
H_ADSCS: High-dose Adipose-derived MSCs
L_BMSCS: Low-dose Bone Marrow-derived Mesenchymal Stem Cells
MSCs: Mesenchymal Stem Cells
NMA: Network Meta-analysis
RCTs: Randomized Controlled Trials
KOA: Knee Osteoarthritis
MD: Mean Difference
OR: Odds Ratio
CI: 95% Confidence Interval
m: Month
y: Year
WOMAC: Western Ontario MacMaster total score
VAS: Visual Analog Scale
AEs: Adverse Events
BMI: Body Mass Index
K-L grade: Kellgren-Lawrence Grading Scale
SUCRA: Surface Under the Cumulative Ranking Curve

## Additional Information and Declarations

### Data Availability

The following information was supplied regarding data availability: The raw data are available in the Supplemental Files.
