# Supplementary material for: Comparative efficacy of different doses of mesenchymal stem cells derived from different tissue sources for knee osteoarthritis: a systematic review and network meta-analysis of randomized controlled trials
Source: PeerJ. 2026 Mar 9;14:e20776. doi: 10.7717/peerj.20776 (PMC12981242; doi:10.7717/peerj.20776)
Supplement: Supplemental Information 8 [file peerj-14-20776-s008.docx]

**Supplementary materials**

**Table S1.** SUCRA RANK

| **Treatment/outcome (%)** | **AEs** | **Womac (6months)** | **Womac (12months)** | **VAS**  **(3months)** | **VAS**  **(6months)** | **VAS**  **(12months)** |
| --- | --- | --- | --- | --- | --- | --- |
| **Control** | 42 | 14.3 | 2.7 | 0.2 | 49.6 | 3.1 |
| **H_ADSCS** | 23 | 54.8 | 73.5 | 83.6 | 39.8 | 52.4 |
| **L_ADSCS** | 85.9 | 66.7 | 75.8 | 83.5 | 25.9 | 67.1 |
| **L_BMSCS** | NR | 35.1 | 45.3 | 41.7 | 58.5 | 44.8 |
| **L_UCSCS** | 29.8 | 44.2 | 26.5 | 17.7 | 67.7 | 19.6 |
| **M_ADSCS** | 74.9 | 58.3 | 99.3 | 82.7 | 17.7 | 73.0 |
| **M_BMSCS** | 28.1 | 74.2 | 26.8 | NR | 60.8 | 68.5 |
| **M_UCSCS** | 65.9 | 52.4 | NR | 40.3 | 80.0 | 71.4 |

**Table S2:** Table of pair-wise

| Comparison (WOMAC 6) | The amount of researches | heterogeneity（i^2^） | MD (95%CI) |
| --- | --- | --- | --- |
| H_ADSCS vs Control | 2 | 96.4% | -18.440 (-44.155,5.7009) |
| H_ADSCS vs Control | 1 | NR | -7.3499 (-46.767,32.280) |
| L_UCSCS vs Control | 1 | NR | -12.899 (-50.703,24.951) |
| M_BMSCS vs Control | 1 | NR | -31.029 (-73.588,10.84) |
| M_UCSCS vs Control | 1 | NR | -16.91 (-29.76,-4.07) |
| L_ADSCS vs H_ADSCS | 2 | 11.2% | -4.57 (-34.08,24.21） |
| M_ADSCS vs L_ADSCS | 2 | 0.0% | -1.88 (-28.42,23.79） |
| M_ADSCS vs L_ADSCS | 2 | 0.0% | 6.52% (-28.83,41.48) |
| M_UCSCS vs L_UCSCS | 1 | NR | -3.62 (-39.60,32.02) |
|  | | | |
| Comparison (WOMAC 12) | The amount of researches | heterogeneity（i^2^） | MD (95%CI) |
| H_ADSCS vs Control | 1 | NR | -37.39 (-74.96,0.11) |
| L_BMSCS vs Control | 1 | NR | -18.96 (-58.21,20.92) |
| L_UCSCS vs Control | 1 | NR | -8.92 (-47.26,29.41) |
| M_BMSCS vs Control | 1 | NR | -8.83 (-48.04,29.81) |
| L_ADSCS vs H_ADSCS | 2 | 58.7% | 1.36 (-29.67,28.58) |
| M_ADSCS vs H_ADSCS | 2 | 34.6% | -12.88 (-39.53,15.60) |
| M_ADSCS vs L_ADSCS | 2 | 20.2% | -0.57 (-38.92,31.92) |
|  | | | |
| Comparison (VAS 3) | The amount of researches | heterogeneity（i^2^） | MD (95%CI) |
| H_ADSCS vs Control | 2 | 81.0% | -13.68 (-29.56,-0.60) |
| L_BMSCS vs Control | 1 | NR | -1.99 (-21.29,17.35) |
| L_UCSCS vs Control | 2 | 0.0% | -0.57 (-14.53,13.09) |
| M_BMSCS vs Control | 1 | NR | -1.88 (-21.40,17.43) |
| L_ADSCS vs H_ADSCS | 1 | NR | -0.07 (-20.02,19.58) |
| M_ADSCS vs H_ADSCS | 1 | NR | 0.01 (-19.37,19.72) |
| M_ADSCS vs L_ADSCS | 1 | NR | -0.04 (-20.08,19.84) |
| M_UCSCS vs L_UCSCS | 1 | NR | -1.14 (-21.13,18.38) |
|  | | | |
| Comparison (VAS 6) | The amount of researches | heterogeneity（i^2^） | MD (95%CI) |
| H_ADSCS vs Control | 2 | 96.2% | -6.13 (-23.18,9.80) |
| L_BMSCS vs Control | 1 | NR | -2.89 (-26.14,20.20) |
| L_UCSCS vs Control | 3 | 99.6% | -4.11（-19.84，8.26） |
| M_BMSCS vs Control | 1 | NR | -5.15（-39.06，28.94） |
| M_UCSCS vs L_UCSCS | 2 | 99.3% | -8.16 (-29.73,7.59) |
| L_ADSCS vs H_ADSCS | 2 | 89.5% | 3.86 (-12.11,22.96) |
| M_ADSCS vs H_ADSCS | 2 | 99.0% | 6.53 (-8.55,27.40) |
| M_ADSCS vs L_ADSCS | 2 | 0.0% | 0.48 (-21.21,22.99) |
| M_UCSCS vs L_UCSCS | 2 | 61.2% | -3.31(-21.73,14.17) |
| M_UCSCS vs Control | 2 | 99.3% | -8.16 (-29.73,7.59) |
| L_ADSCS vs H_ADSCS | 2 | 89.5% | 3.86 (-12.11,22.96) |
| M_ADSCS vs H_ADSCS% | 2 | 99.0% | 6.53 (-8.55,27.40) |
| M_ADSCS vs L_ADSCS | 2 | 0.0% | 0.48 (-21.21,22.99) |
| M_UCSCS vs L_UCSCS | 2 | 61.2% | -3.31 (-21.73,14.17) |
| M_UCSCS vs L_UCSCS | 2 | 61.2% | -3.31 (-21.72,14.17) |
| H_ADSCS vs Control | 1 | NR | -3.91 (-12.61,4.80) |
| L_BMSCS vs Control | 1 | NR | -3.21 (-12.19,5.72) |
| L_UCSCS vs Control | 2 | 53.9% | -1.48 (-7.68,4.72) |
| M_BMSCS vs Control | 1 | NR | -8.08 (-29.38,12.62) |
| M_UCSCS vs Control | 1 | NR | -4.58 (-13.27,4.09) |
| L_ADSCS vs H_ADSCS | 2 | 43.5% | 0.11 (-5.70,10.12) |
| M_ADSCS vs H_ADSCS | 2 | 47.1% | -0.50 (-6.03,10.25) |
| M_ADSCS vs L_ADSCS | 2 | 0.0% | -0.15 (-8.66,9.01) |
| M_UCSCS vs L_UCSCS | 2 | 0.0% | -2.89 (-10.92,3.90) |
|  | | | |
| Comparison (VAS 12) | The amount of researches | heterogeneity（i^2^） | MD (95%CI) |
| H_ADSCS vs Control | 1 | NR | -3.90 (-12.61,4.80) |
| L_BMSCS vs Control | 1 | NR | -3.21 (-12.19.5.72) |
| L_UCSCS vs Control | 2 | 53.9% | -1.48 (-7.68,4.72) |
| M_BMSCS vs Control | 1 | NR | -8.08 (-29.69,13.10) |
| M_UCSCS vs Control | 1 | NR | -4.57 (-13.27,4.09) |
| L_ADSCS vs H_ADSCS | 2 | 43.5% | 0.11 (-5.70,10.12) |
| M_ADSCS vs L_ADSCS | 2 | 0.0% | -0.15 (-8.66,9.01) |
| M_ADSCS vs H_ADSCS | 2 | 47.1% | -0.50 (-6.03,10.25) |
| M_UCSCS vs L_UCSCS | 2 | 0.0% | -2.89 (-10.92,3.90) |
|  | | | |
| Comparison (AE) | The amount of researches | heterogeneity（i^2^） | OR (95%CI) |
| H_ADSCS vs Control | 2 | 99.0% | 17.73 (0.0008,9.35) |
| L_UCSCS vs Control | 2 | 0.0% | 1.09 (9.04e-08,2.02e+06) |
| M_BMSCS vs Control | 1 | NR | 1.83 (1.30e-07,2.48e+07) |
| M_UCSCS vs Control | 1 | NR | 2.68e-05 (3.49e-26,6.05e+16） |
| L_ADSCS vs H_ADSCS | 1 | NR | 2.95e-06 (3.49e-18,2.13e+06) |
| M_ADSCS vs H_ADSCS | 1 | NR | 5.60e-06 (3.20e-20,1.03e+09) |
| M_ADSCS vs L_ADSCS | 1 | NR | 3.93 (2.43e-07,8.68e+07) |
| M_UCSCS vs L_UCSCS | 1 | NR | 0.81 (3.17e-70,1.98e+67) |

**
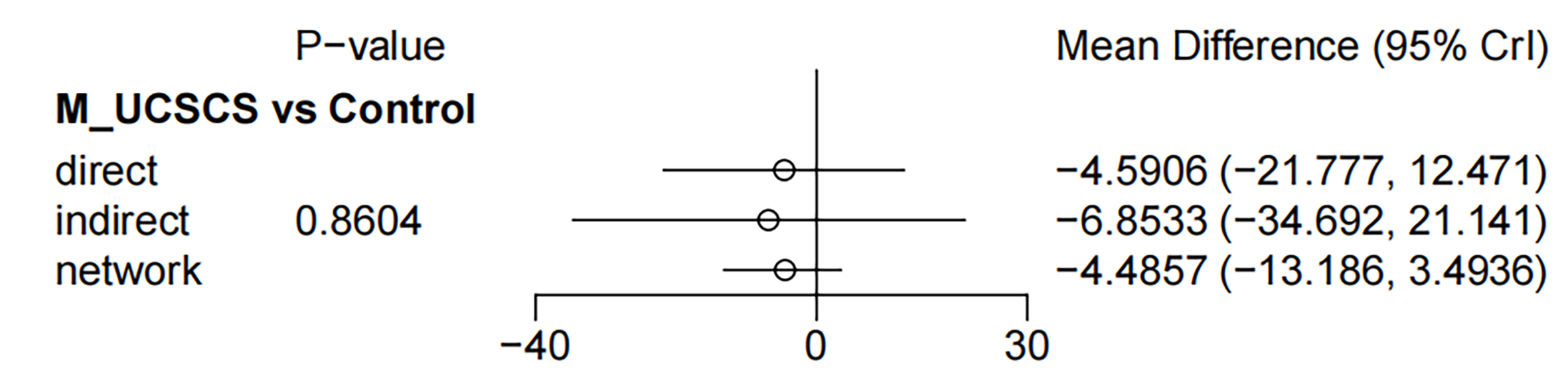
**

**Figure S1:** Diagram of local inconsistency comparison of long-term VAS

**
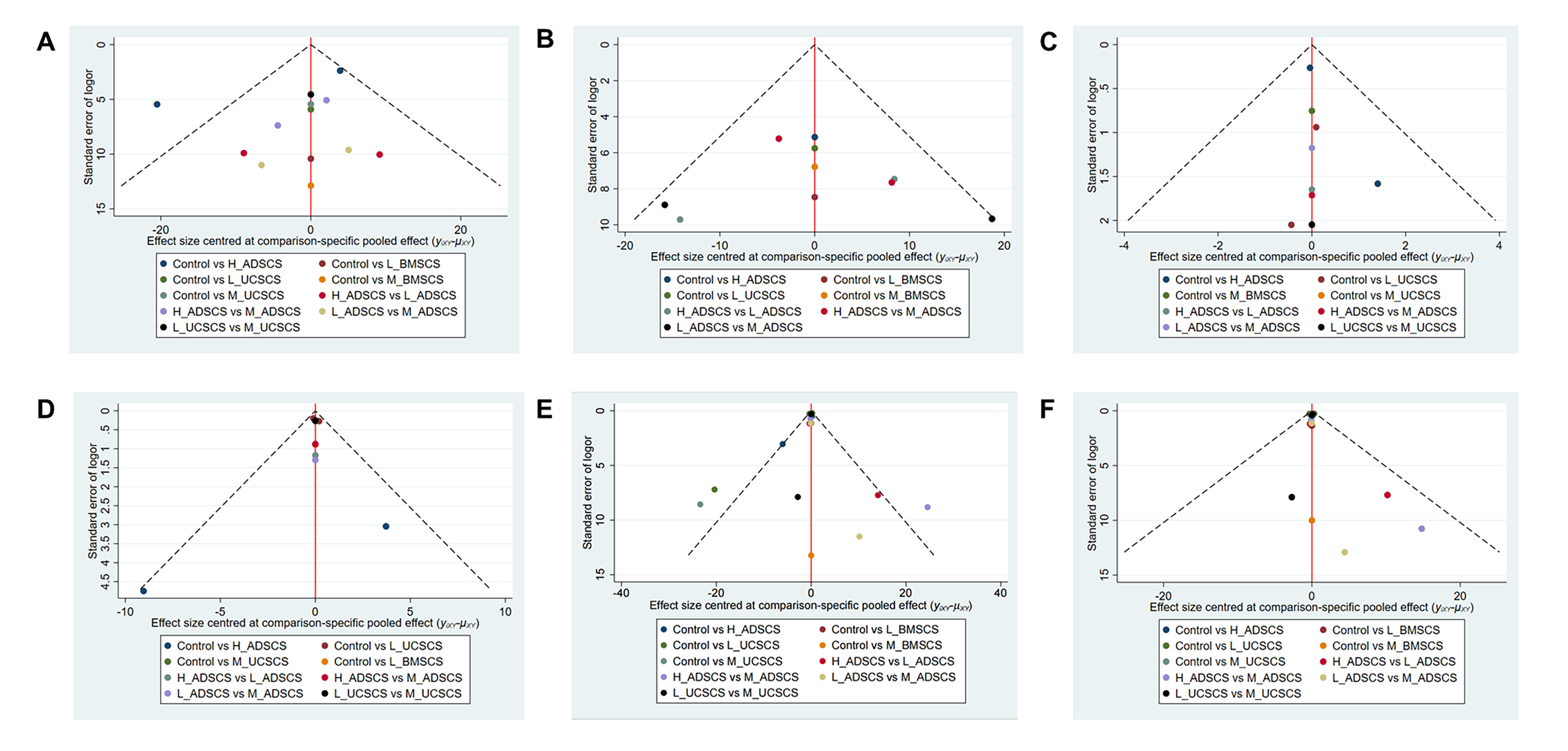
**

**Figure S2:** Funnel plots

Funnel plots of WOMAC (6 months); B. Funnel plots of WOMAC (12 months); C. Funnel plots of Adverse Events; D. Funnel plots of VAS (3 months); E. Funnel plots of VAS (6 months); F: Funnel plots of VAS (12 months)

**Supplementary Appendix 1** Search strategy

**Pubmed**

#1 "Osteoarthritis"[Title/Abstract] OR "Osteoarthritides"[Title/Abstract] OR "Osteoarthrosis"[Title/Abstract] OR "Osteoarthroses"[Title/Abstract] OR "Degenerative Arthritides"[Title/Abstract] OR "Degenerative Arthritis"[Title/Abstract] OR "Arthrosis"[Title/Abstract] OR "Arthroses"[Title/Abstract] OR "Osteoarthrosis Deformans"[Title/Abstract] OR "OA"[Title/Abstract] OR "Osteoarthritic"[Title/Abstract]

#2 "Osteoarthritis"[Mesh]

#3 "Knee"[Title/Abstract] OR "knees"[Title/Abstract]

#4 "Knee"[Mesh]

#5 "Mesenchymal Stem Cells"[Title/Abstract] OR "Mesenchymal Stem Cell"[Title/Abstract] OR "Bone Marrow Mesenchymal Stem Cells"[Title/Abstract] OR "Bone Marrow Mesenchymal Stem Cell"[Title/Abstract] OR "Bone Marrow Stromal Cells"[Title/Abstract] OR "Bone Marrow Stromal Cell"[Title/Abstract] OR "Multipotent Bone Marrow Stromal Cell"[Title/Abstract] OR "Multipotent Bone Marrow Stromal Cells"[Title/Abstract] OR "Adipose Derived Mesenchymal Stem Cells"[Title/Abstract] OR "Adipose Derived Mesenchymal Stromal Cells"[Title/Abstract] OR "Adipose Derived Mesenchymal Stem Cell"[Title/Abstract] OR "Adipose Tissue Derived Mesenchymal Stem Cell"[Title/Abstract] OR "Adipose Tissue Derived Mesenchymal Stem Cells"[Title/Abstract] OR "Adipose Tissue Derived Mesenchymal Stromal Cells"[Title/Abstract] OR "Adipose Tissue Derived Mesenchymal Stromal Cell"[Title/Abstract] OR "Mesenchymal Stromal Cells"[Title/Abstract] OR "Mesenchymal Stromal Cell"[Title/Abstract] OR "Multipotent Mesenchymal Stromal Cells"[Title/Abstract] OR "Multipotent Mesenchymal Stromal Cell"[Title/Abstract] OR "Mesenchymal Progenitor Cell"[Title/Abstract] OR "Mesenchymal Progenitor Cells"[Title/Abstract] OR "Wharton Jelly Cells"[Title/Abstract] OR "Whartons Jelly Cells"[Title/Abstract] OR "Bone Marrow Stromal Stem Cells"[Title/Abstract] OR "Bone Marrow Stromal Stem Cell"[Title/Abstract] OR "Stem cell"[Title/Abstract] OR "Stem cells"[Title/Abstract] OR "Stromal cells"[Title/Abstract] OR "Stromal cell"[Title/Abstract] OR "MSC"[Title/Abstract] OR "Progenitor Cell"[Title/Abstract] OR "Progenitor Cells"[Title/Abstract] OR " Synovial stem cells"[Title/Abstract] OR " synovium-derived MSCs"[Title/Abstract] OR " Synovial-derived mesenchymal stem cells"[Title/Abstract]

#6 Mesenchymal Stem Cells[Mesh Terms]

#7 (#1 OR #2) AND (#3 OR #4) AND (#5 OR #6)

**Web of Science**

#1 TS=((Osteoarthritis OR Osteoarthritides OR Osteoarthrosis OR Osteoarthroses OR Degenerative Arthritides OR Degenerative Arthritis OR Arthrosis OR Arthroses OR Osteoarthrosis Deformans OR OA OR Osteoarthritic))

#2 TS=((knee OR knees))

#3 TS=((Mesenchymal Stem Cells OR Mesenchymal Stem Cell OR Bone Marrow Mesenchymal Stem Cells OR Bone Marrow Mesenchymal Stem Cell OR Bone Marrow Stromal Cells OR Bone Marrow Stromal Cell OR Multipotent Bone Marrow Stromal Cell OR Multipotent Bone Marrow Stromal Cells OR Adipose Derived Mesenchymal Stem Cells OR Adipose Derived Mesenchymal Stromal Cells OR Adipose Derived Mesenchymal Stem Cell OR Adipose Tissue Derived Mesenchymal Stem Cell OR Adipose Tissue Derived Mesenchymal Stem Cells OR Adipose Tissue Derived Mesenchymal Stromal Cells OR Adipose Tissue Derived Mesenchymal Stromal Cell OR Mesenchymal Stromal Cells OR Mesenchymal Stromal Cell OR Multipotent Mesenchymal Stromal Cells OR Multipotent Mesenchymal Stromal Cell OR Mesenchymal Progenitor Cell OR Mesenchymal Progenitor Cells OR Wharton Jelly Cells OR Whartons Jelly Cells OR Bone Marrow Stromal Stem Cells OR Bone Marrow Stromal Stem Cell OR Stem cell OR Stem cells OR Stromal cells OR Stromal cell OR MSC OR Progenitor Cell OR Progenitor Cells OR Synovial stem cells OR synovium-derived MSCs OR Synovial-derived mesenchymal stem cells))

#4 #1 AND #2 AND #3

**CochraneLibrary**

#1 MeSH descriptor: [Osteoarthritis] explode all trees

#2 (Osteoarthritis):ti,ab,kw OR (Osteoarthritides):ti,ab,kw OR (Osteoarthrosis):ti,ab,kw OR (Osteoarthroses):ti,ab,kw OR (Degenerative Arthritides):ti,ab,kw OR (Degenerative Arthritis):ti,ab,kw OR (Arthrosis):ti,ab,kw OR (Arthroses):ti,ab,kw OR (Osteoarthrosis Deformans):ti,ab,kw OR (OA):ti,ab,kw OR (Osteoarthritic):ti,ab,kw

#3 #1 OR #2

#4 MeSH descriptor: [Mesenchymal Stem Cells] explode all trees

#5 (Mesenchymal Stem Cells):ti,ab,kw OR (Mesenchymal Stem Cell):ti,ab,kw OR (Bone Marrow Mesenchymal Stem Cells):ti,ab,kw OR (Bone Marrow Mesenchymal Stem Cell):ti,ab,kw OR (Bone Marrow Stromal Cells):ti,ab,kw OR (Bone Marrow Stromal Cell):ti,ab,kw OR (Multipotent Bone Marrow Stromal Cell):ti,ab,kw OR (Multipotent Bone Marrow Stromal Cells):ti,ab,kw OR (Adipose Derived Mesenchymal Stem Cells):ti,ab,kw OR (Adipose Derived Mesenchymal Stromal Cells):ti,ab,kw OR (Adipose Derived Mesenchymal Stem Cell):ti,ab,kw OR (Adipose Tissue Derived Mesenchymal Stem Cell):ti,ab,kw OR (Adipose Tissue Derived Mesenchymal Stem Cells):ti,ab,kw OR (Adipose Tissue Derived Mesenchymal Stromal Cells):ti,ab,kw OR (Adipose Tissue Derived Mesenchymal Stromal Cell):ti,ab,kw OR (Mesenchymal Stromal Cells):ti,ab,kw OR (Mesenchymal Stromal Cell):ti,ab,kw OR (Multipotent Mesenchymal Stromal Cells):ti,ab,kw OR (Multipotent Mesenchymal Stromal Cell):ti,ab,kw OR (Mesenchymal Progenitor Cell):ti,ab,kw OR (Mesenchymal Progenitor Cells):ti,ab,kw OR (Wharton Jelly Cells):ti,ab,kw OR (Whartons Jelly Cells):ti,ab,kw OR (Bone Marrow Stromal Stem Cells):ti,ab,kw OR (Bone Marrow Stromal Stem Cell):ti,ab,kw OR (Stem cell):ti,ab,kw OR (Stem cells):ti,ab,kw OR (Stromal cells):ti,ab,kw OR (Stromal cell):ti,ab,kw OR (MSC):ti,ab,kw OR (Progenitor Cell):ti,ab,kw OR (Progenitor Cells):ti,ab,kw OR (Synovial stem cells):ti,ab,kw OR (synovium-derived MSCs):ti,ab,kw OR (Synovial-derived mesenchymal stem cells):ti,ab,kw

#6 #4 OR #5

#7 MeSH descriptor: [Knee] explode all trees

#8 (knee):ti,ab,kw OR (knees):ti,ab,kw

#9 #7 OR #8

#10 #3 AND #6 AND #9

**Embase**

#1 'osteoarthritis':ti,ab,kw OR 'osteoarthritides':ti,ab,kw OR 'osteoarthrosis':ti,ab,kw OR 'osteoarthroses':ti,ab,kw OR 'degenerative arthritides':ti,ab,kw OR 'degenerative arthritis':ti,ab,kw OR 'arthrosis':ti,ab,kw OR 'arthroses':ti,ab,kw OR 'osteoarthrosis deformans':ti,ab,kw OR 'oa':ti,ab,kw OR 'osteoarthritic':ti,ab,kw

#2 'osteoarthritis'/exp

#3 'mesenchymal stem cells':ti,ab,kw OR 'mesenchymal stem cell':ti,ab,kw OR 'bone marrow mesenchymal stem cells':ti,ab,kw OR 'bone marrow mesenchymal stem cell':ti,ab,kw OR 'bone marrow stromal cells':ti,ab,kw OR 'bone marrow stromal cell':ti,ab,kw OR 'multipotent bone marrow stromal cell':ti,ab,kw OR 'multipotent bone marrow stromal cells':ti,ab,kw OR 'adipose derived mesenchymal stem cells':ti,ab,kw OR 'adipose derived mesenchymal stromal cells':ti,ab,kw OR 'adipose derived mesenchymal stem cell':ti,ab,kw OR 'adipose tissue derived mesenchymal stem cell':ti,ab,kw OR 'adipose tissue derived mesenchymal stem cells':ti,ab,kw OR 'adipose tissue derived mesenchymal stromal cells':ti,ab,kw OR 'adipose tissue derived mesenchymal stromal cell':ti,ab,kw OR 'mesenchymal stromal cells':ti,ab,kw OR 'mesenchymal stromal cell':ti,ab,kw OR 'multipotent mesenchymal stromal cells':ti,ab,kw OR 'multipotent mesenchymal stromal cell':ti,ab,kw OR 'mesenchymal progenitor cell':ti,ab,kw OR 'mesenchymal progenitor cells':ti,ab,kw OR 'wharton jelly cells':ti,ab,kw OR 'whartons jelly cells':ti,ab,kw OR 'bone marrow stromal stem cells':ti,ab,kw OR 'bone marrow stromal stem cell':ti,ab,kw OR 'stem cell':ti,ab,kw OR 'stem cells':ti,ab,kw OR 'stromal cells':ti,ab,kw OR 'stromal cell':ti,ab,kw OR 'msc':ti,ab,kw OR 'progenitor cell':ti,ab,kw OR 'progenitor cells':ti,ab,kw OR 'synovial stem cells':ti,ab,kw OR 'synovium-derived mscs':ti,ab,kw OR 'synovial-derived mesenchymal stem cells':ti,ab,kw

#4 'mesenchymal stem cells'/exp

#5 'knee':ti,ab,kw OR 'knees':ti,ab,kw

#6 'knee'/exp

#7 (#1 OR #2) AND (#3 OR #4) AND (#5 OR #6)

**Supplementary Appendix 2** Bias in the each comparisons:

**Primary outcome:**

**Long-term WOMAC(12 months):**


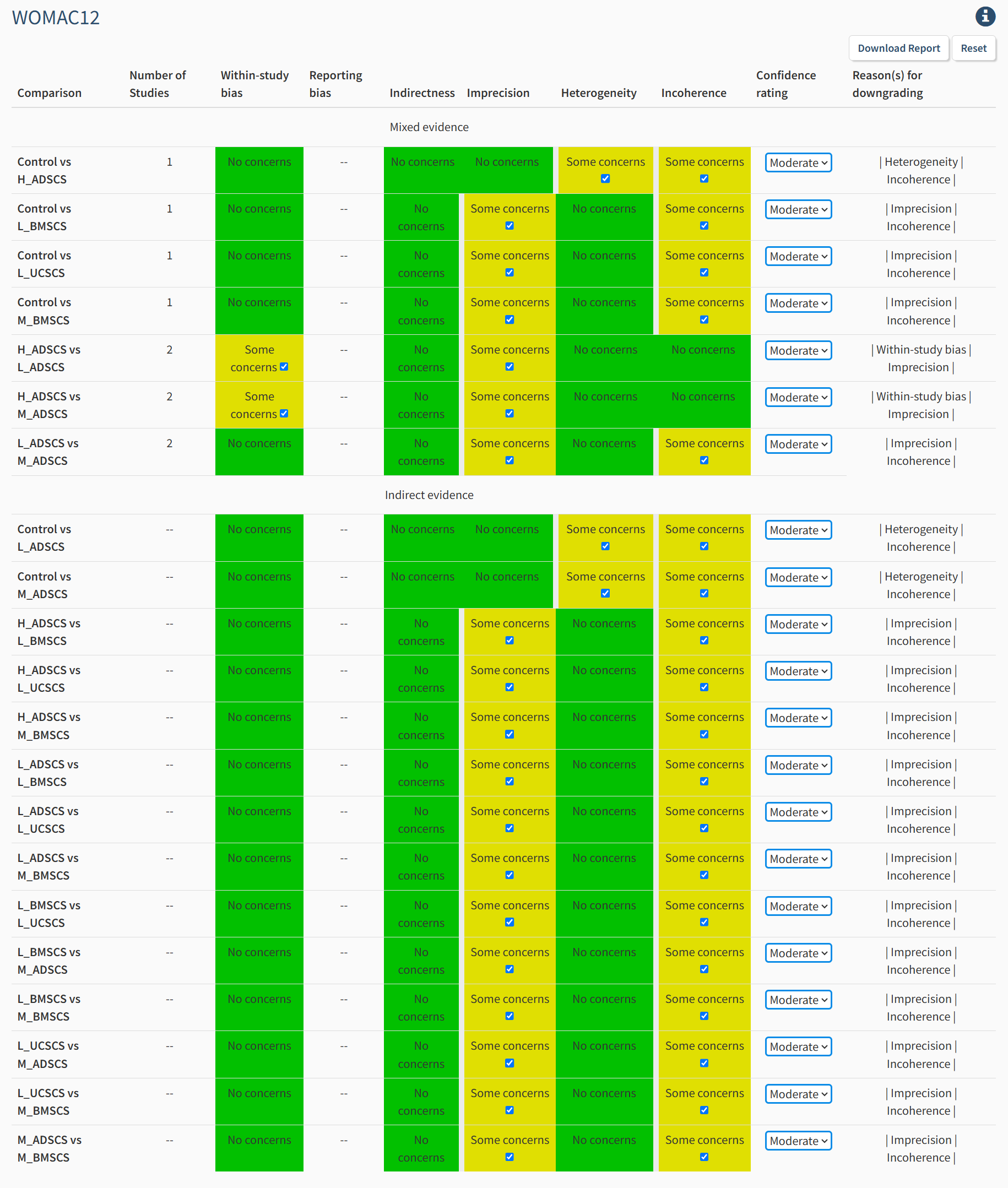


**Long-term VAS(12 months):**


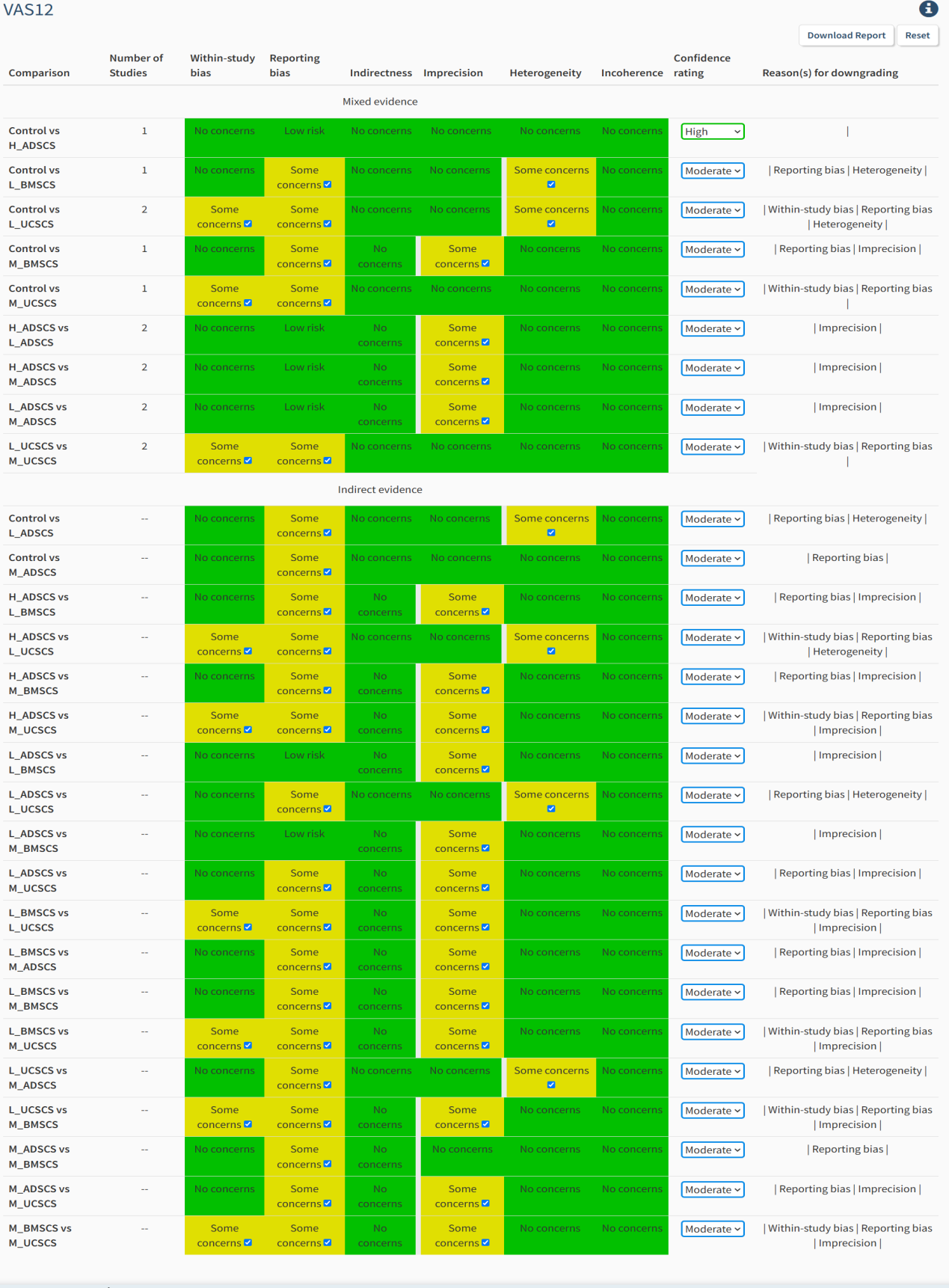


**Secondary Outcome:**

**Medium-term WOMAC (6 months):**

**
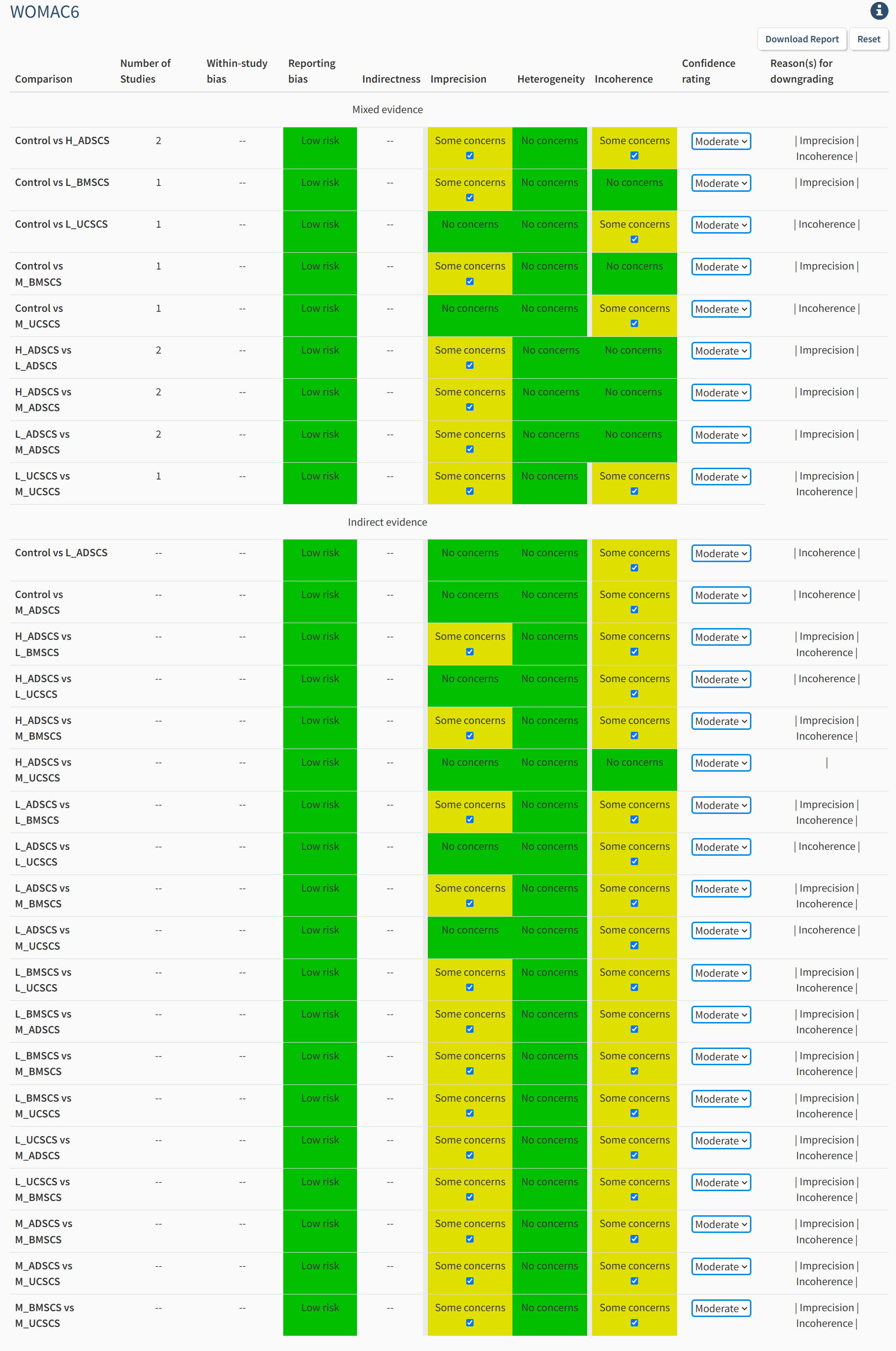
**

**Short-term VAS (3 months):**

**
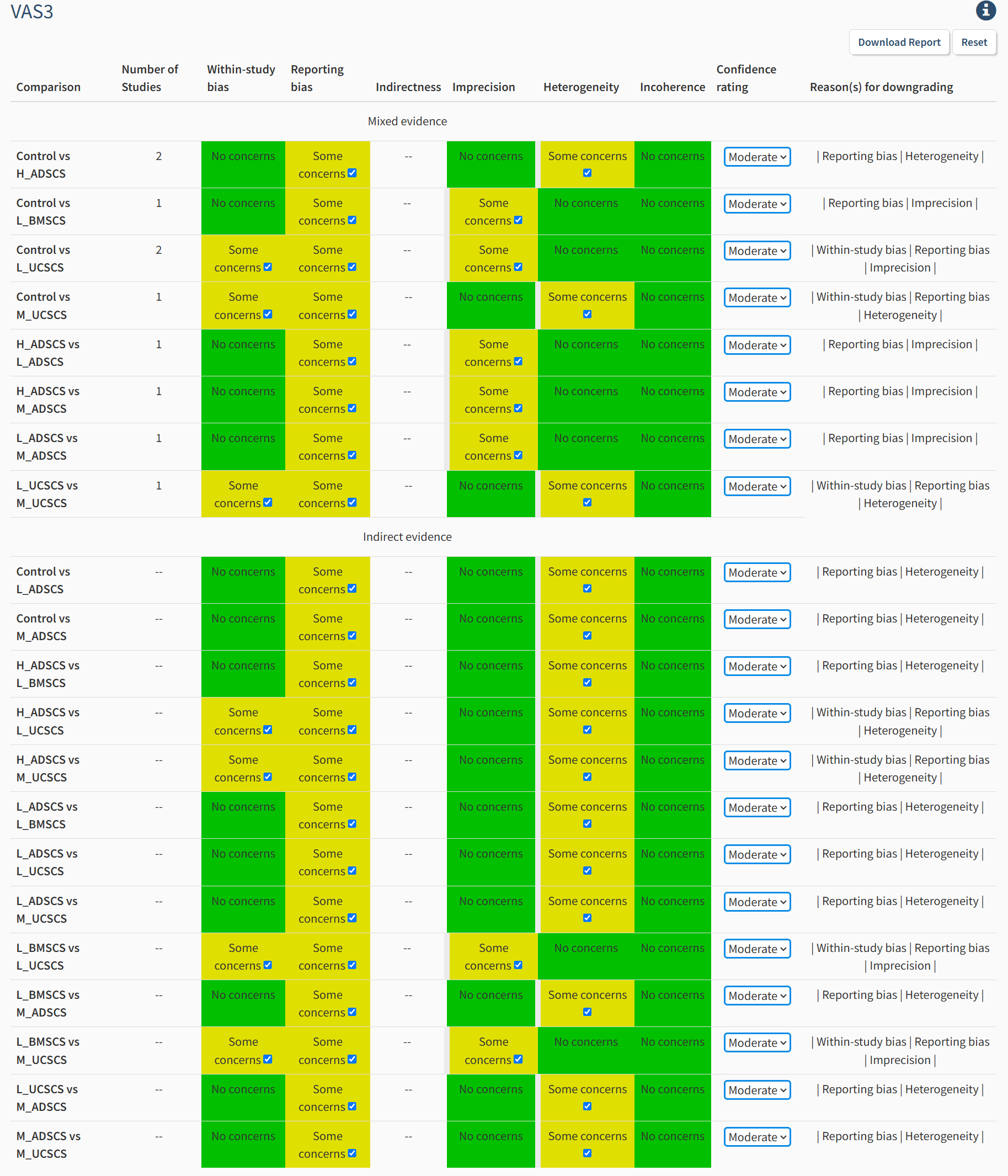
**

**Medium-term VAS (6 months):**

**
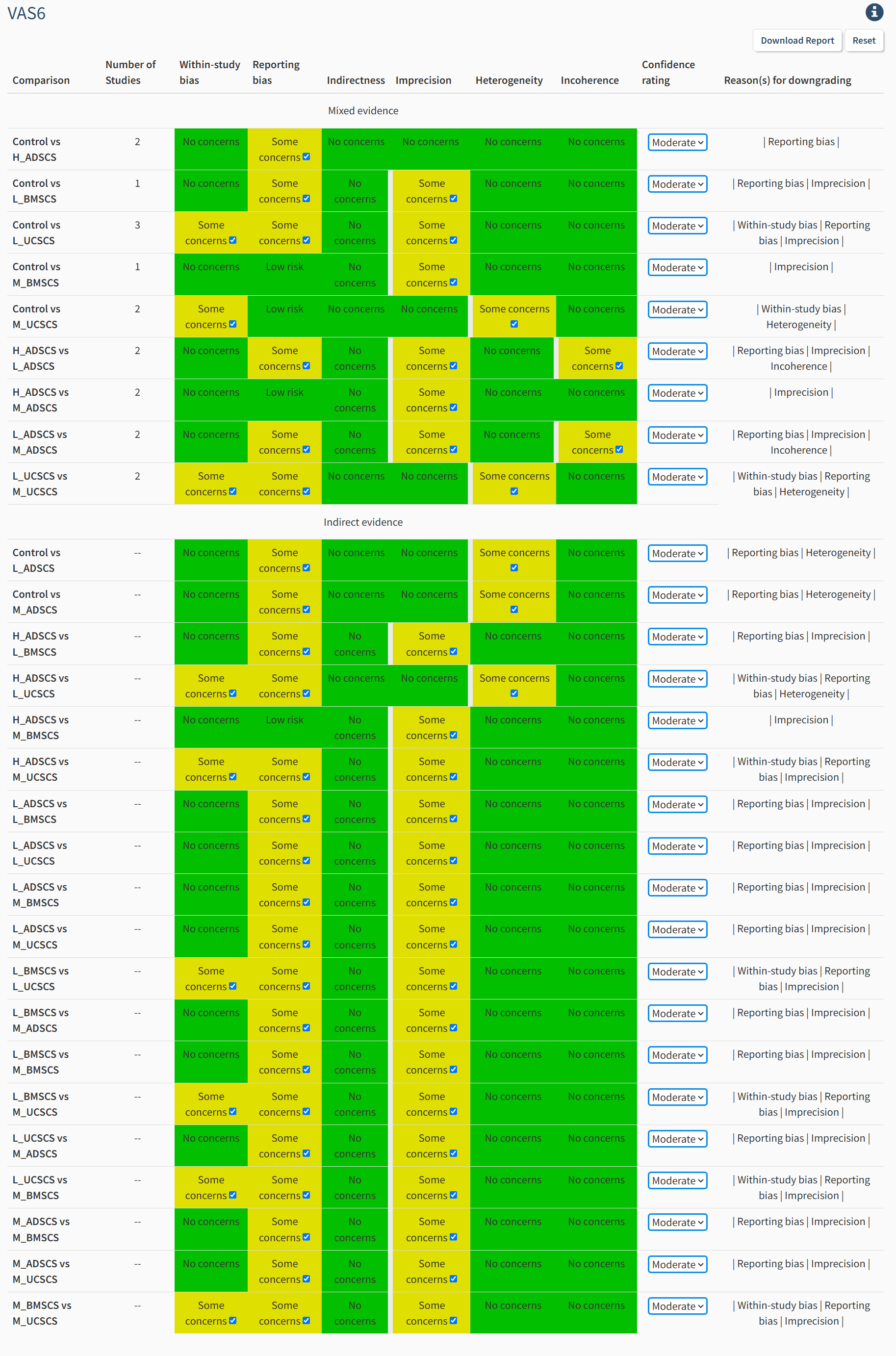
**

**AEs:**

**
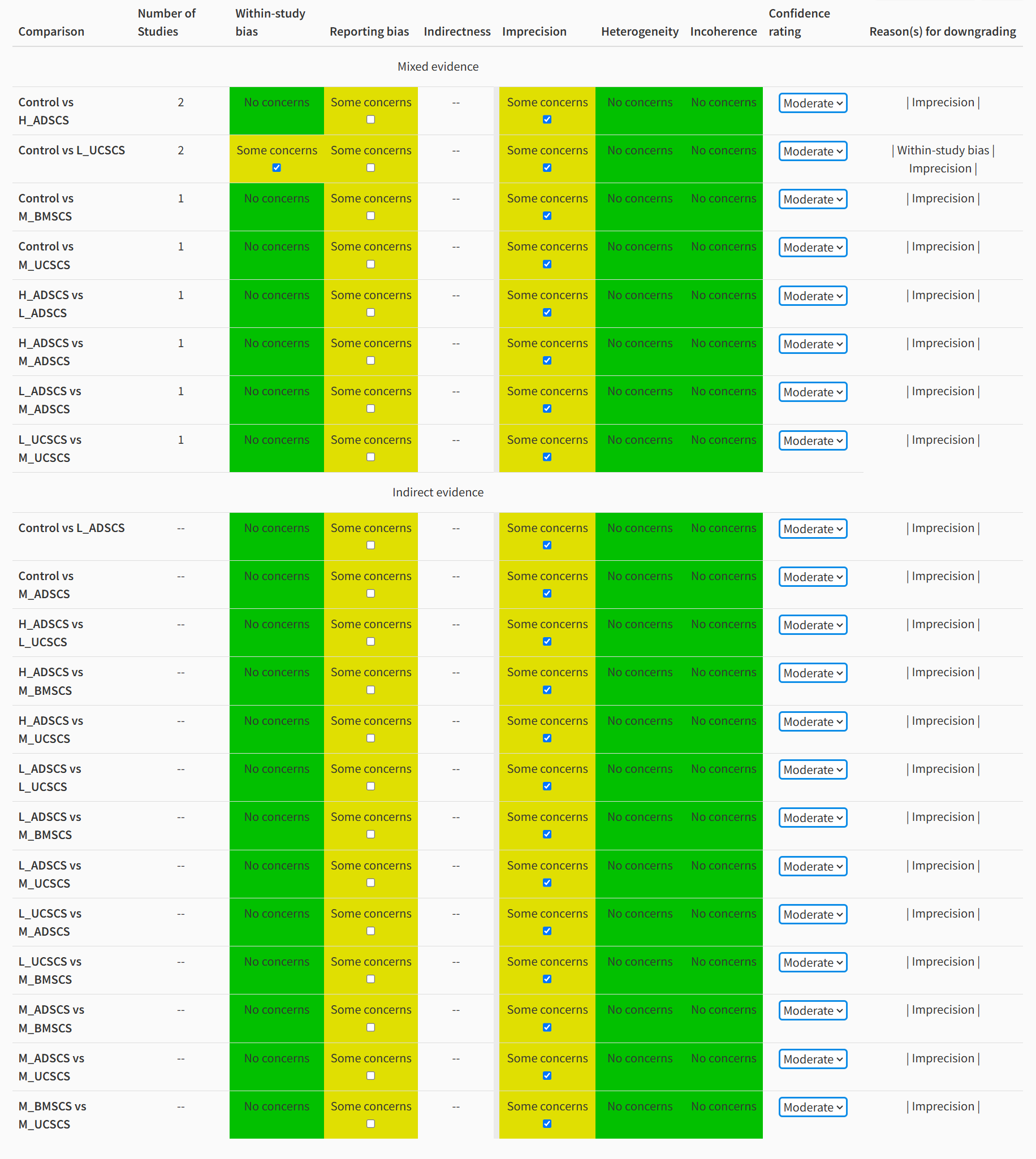
**
